# High amino acid osmotrophic incorporation by marine eukaryotic phytoplankton revealed by click chemistry

**DOI:** 10.1093/ismeco/ycae004

**Published:** 2024-01-17

**Authors:** Catalina Mena, Ona Deulofeu-Capo, Irene Forn, Júlia Dordal-Soriano, Yulieth A Mantilla-Arias, Iván P Samos, Marta Sebastián, Clara Cardelús, Ramon Massana, Cristina Romera-Castillo, Rebeca Mallenco-Fornies, Josep M Gasol, Clara Ruiz-González

**Affiliations:** Department of Marine Biology and Oceanography, Institut de Ciències del Mar (ICM-CSIC), Barcelona 08003, Spain; Department of Marine Biology and Oceanography, Institut de Ciències del Mar (ICM-CSIC), Barcelona 08003, Spain; Department of Marine Biology and Oceanography, Institut de Ciències del Mar (ICM-CSIC), Barcelona 08003, Spain; Department of Marine Biology and Oceanography, Institut de Ciències del Mar (ICM-CSIC), Barcelona 08003, Spain; Department of Marine Biology and Oceanography, Institut de Ciències del Mar (ICM-CSIC), Barcelona 08003, Spain; Department of Marine Biology and Oceanography, Institut de Ciències del Mar (ICM-CSIC), Barcelona 08003, Spain; Department of Marine Biology and Oceanography, Institut de Ciències del Mar (ICM-CSIC), Barcelona 08003, Spain; Department of Marine Biology and Oceanography, Institut de Ciències del Mar (ICM-CSIC), Barcelona 08003, Spain; Department of Marine Biology and Oceanography, Institut de Ciències del Mar (ICM-CSIC), Barcelona 08003, Spain; Department of Marine Biology and Oceanography, Institut de Ciències del Mar (ICM-CSIC), Barcelona 08003, Spain; Department of Marine Biology and Oceanography, Institut de Ciències del Mar (ICM-CSIC), Barcelona 08003, Spain; Department of Marine Biology and Oceanography, Institut de Ciències del Mar (ICM-CSIC), Barcelona 08003, Spain; Department of Marine Biology and Oceanography, Institut de Ciències del Mar (ICM-CSIC), Barcelona 08003, Spain

**Keywords:** osmotrophy, mixotrophy, eukaryotic communities, diatoms, BONCAT, HPG, click chemistry, marine ecology, single-cell microbiology

## Abstract

The osmotrophic uptake of dissolved organic compounds in the ocean is considered to be dominated by heterotrophic prokaryotes, whereas the role of planktonic eukaryotes is still unclear. We explored the capacity of natural eukaryotic plankton communities to incorporate the synthetic amino acid L-homopropargylglycine (HPG, analogue of methionine) using biorthogonal noncanonical amino acid tagging (BONCAT), and we compared it with prokaryotic HPG use throughout a 9-day survey in the NW Mediterranean. BONCAT allows to fluorescently identify translationally active cells, but it has never been applied to natural eukaryotic communities. We found a large diversity of photosynthetic and heterotrophic eukaryotes incorporating HPG into proteins, with dinoflagellates and diatoms showing the highest percentages of BONCAT-labelled cells (49 ± 25% and 52 ± 15%, respectively). Among them, pennate diatoms exhibited higher HPG incorporation in the afternoon than in the morning, whereas small (≤5 μm) photosynthetic eukaryotes and heterotrophic nanoeukaryotes showed the opposite pattern. Centric diatoms (e.g. *Chaetoceros*, *Thalassiosira*, and *Lauderia* spp*.*) dominated the eukaryotic HPG incorporation due to their high abundances and large sizes, accounting for up to 86% of the eukaryotic BONCAT signal and strongly correlating with bulk ^3^H-leucine uptake rates. When including prokaryotes, eukaryotes were estimated to account for 19–31% of the bulk BONCAT signal. Our results evidence a large complexity in the osmotrophic uptake of HPG, which varies over time within and across eukaryotic groups and highlights the potential of BONCAT to quantify osmotrophy and protein synthesis in complex eukaryotic communities.

## Introduction

The consumption of dissolved organic matter (DOM) in the ocean is considered to be dominated by heterotrophic prokaryotes [1, 2], which are more efficient than eukaryotes in taking up the diluted organic compounds in natural marine environments [3, 4]. However, studies based on monospecific cultures of eukaryotic phytoplankton have repeatedly shown their ability to take up a variety of organic substrates, such as amino acids, glucose, acetate, mannitol, glycerol, urea, and humic acids, among others [5–11], and there are evidences that these microorganisms are capable of consuming dissolved organic substrates at equally low concentrations as prokaryotes [6, 9]. Recently, genomic and transcriptomic surveys have also provided insight into the trophic flexibility of marine algae and their use of organic compounds as nutrient source [12–14]. While all these evidences that phytoplankton osmotrophy, understood here as the use of organic substrates to obtain carbon and noncarbon elements beyond auxotrophy [5, 15], is a common strategy among phytoplankton groups, the ecological relevance of this process, its drivers, and spatio-temporal variability remain poorly understood, limiting our comprehension of organic matter flows in the ocean.

In recent years, there has been an increasing awareness of the importance of the different forms of mixotrophy (autotrophy + heterotrophy) among protist plankton [5, 16], but these efforts have focused mostly on phagotrophy. Studies focusing on phytoplankton osmotrophy have suggested this process to be a survival strategy under limited nutrient or light conditions, serving as carbon and nitrogen source [14, 17–19], but its regulation remains unclear. Some osmotrophs are able to switch between photosynthesis and heterotrophy depending on the light conditions and organic or inorganic resource availability [20]. However, light can affect phytoplankton osmotrophy either positively or negatively depending on the light levels, the organic compound, and the species considered [9, 21–24], in turn modulating the relative use of organic and inorganic compounds [10, 19, 25]. Consequently, it is not clear under which circumstances osmotrophy may represent a competitive advantage for certain eukaryotes and whether it may channel significant amounts of organic matter compared to prokaryotic heterotrophy.

The bulk osmotrophic activity of natural eukaryotic communities [10, 19, 21, 23, 26] or monospecific cultures [27–29] has been mostly quantified using radiolabelled or stable isotope labelled compounds. At the single-cell level, the uptake of organic compounds has been traced mainly through microautoradiography [19, 21, 24, 25, 30], a technique that allows the identification through microscopy of individual cells active in the uptake of specific radiolabelled substrates and through nanoSIMS [31], which analyses the isotopic composition of labelled cells. These studies have evidenced that not all taxa or cells within communities are active or equally active, suggesting that depending on the community composition the osmotrophic capacity of phytoplankton assemblages may vary. However, the complexity, time-consuming and expensive nature of microautoradiography or nanoSIMS have discouraged an intense use of these techniques for the study of eukaryotic osmotrophy. Click chemistry-based approaches like biorthogonal noncanonical amino acid tagging (BONCAT) [32, 33] have recently emerged as a promising alternative to visually identify translationally active microbial cells [34-36]. BONCAT uses synthetic amino acids (analogues for methionine) that when incorporated into cells can be fluorescently detected via copper-catalyzed alkyne–azide click chemistry. This method has the advantage of detecting active substrate incorporation and allocation of protein translation without altering cellular physiology [37, 38]. Moreover, the fluorescence intensity of the BONCAT signal correlates well with measured prokaryotic heterotrophic production rates [35] and hence has been mostly applied to heterotrophic prokaryotic communities [34, 35, 39–42]. Its use in planktonic eukaryotes has been limited to a few cultures of *Emiliana huxleyi* [43], *Cafeteria burkardae* [44], and *Ostreococcus* sp. and *Micromonas pusilla* [38] that have successfully shown uptake of the BONCAT substrates. To our knowledge, however, no studies have used BONCAT to investigate the contribution of different taxa to osmotrophic activity and its short-term variability within complex eukaryotic phytoplankton communities.

Here, we explored the incorporation of a methionine analogue by individual eukaryotic cells within natural communities and its short-term changes using the BONCAT method in a coastal Mediterranean site. The aims of this study were to (i) test the potential of BONCAT to identify active eukaryotes and quantify the contribution to osmotrophic activity by different phytoplankton groups; (ii) evaluate the short-term changes in osmotrophic activity and their potential drivers; (iii) estimate the eukaryotic versus prokaryotic contribution to total substrate incorporation; and (iv) explore whether any of the BONCAT-positive eukaryotes correlate with bulk ^3^H-leucine incorporation, an independent measure of osmotrophic activity commonly attributed to prokaryotes. To assess these goals, we analysed the BONCAT-based activity of different eukaryotic groups over a 9-day survey, comparing morning and afternoon day times, and explored the environmental drivers of the observed variability. Flow cytometry data and sequencing of the 18S rRNA gene were used for the quantification and identification of the most important phytoplankton groups at the site, and the bulk community heterotrophic activity was measured as ^3^H-leucine incorporation. The results provide new insights into the role of protists as key DOM consumers in marine ecosystems.

## Materials and methods

### Field sampling and basic parameters

Sampling was carried out from 8 to 16 February 2021 at the Blanes Bay Microbial Observatory, a coastal station 1 km offshore in the NW Mediterranean (41°39.90’N, 2°48.03′E). Surface water samples (0.5 m depth) were collected in polycarbonate carboys twice a day during 9 days, in the morning (at 10:00 h, 2 h after dawn) and afternoon (at 17:00 h, 1 h before dusk), to explore the short-term changes in HPG uptake during the daylight hours when photosynthesis takes place. Samples were transported to the laboratory in the dark and all incubations started less than 3.5 h after water collection. The afternoon sample of 14 February could not be collected because of rough sea conditions.

Temperature, salinity and turbidity of the sampled waters were obtained with a SAIV A-S 204 conductivity–temperature–depth probe. Solar irradiance data were obtained from the automatic weather station on-land at Malgrat de Mar, close to the sampling site (http://www.meteo.cat). Inorganic nutrient concentrations were analysed using an AA3 HR autoanalyser (Seal Analytical). Chlorophyll-a concentration was determined in triplicate and extracted in acetone (90% v:v). Samples for total organic carbon (TOC) were collected in precombusted glass vials and measured on a Shimatzu TOC-V analyser. Fluorescent dissolved organic matter (FDOM) was measured once per day to monitor the organic matter quality. FDOM samples were filtered through precombusted GF/F filters and were measured using a LS55 Perkin Elmer Luminescence spectrometer following Romera-Castillo *et al*. [42]. Peaks FDOM-C, FDOM-A, and FDOM-M are associated with humic-like substances while peaks FDOM-T and FDOM-B correspond to protein-like substances [45].

### Prokaryotic abundance and bulk ^3^H-leucine incorporation rates

The abundance of heterotrophic and phototrophic (*Prochlorococcus* and *Synechococcus*) prokaryotes was measured using flow cytometry as described in Gasol and Morán [46] using an AccuryC6 Plus and FACSCalibur (Becton Dickinson). Bulk incorporation of tritiated leucine was estimated following Kirchman *et al*. [47] using the processing method of Smith and Azam [48].

### Characterization of eukaryotic communities

Water samples (10 l) of each time point prefiltered by 20 μm were sequentially filtered through 3 and 0.2 μm pore-size filters. DNA was extracted from the 0.2 to 3 and the 3 to 20 μm size fractions with phenol-chloroform as described elsewhere [49]. 18S rRNA gene amplification of the V4 region was performed using Balzano *et al*. [50] primers, and PCR products were sequenced using a NovaSeq PE250 (Illumina). Raw reads were processed with DADA2 v1.12.1 [51] and taxonomically classified with the eukaryotes V4 database [52].

### Single-cell eukaryotic and prokaryotic activities through biorthogonal noncanonical amino acid tagging click chemistry

The osmotrophic activity was estimated using BONCAT following the protocol described in Leizeaga *et al*. [35] with some modifications. 90- and 9-ml samples for eukaryotes and prokaryotes, respectively, were incubated during 2 h with the synthetic amino acid L-homopropargylglycine (HPG, methionine analogue) at 2 μM final concentration at *in situ* temperature in the dark. The incubation time and HPG concentration were chosen based on the method optimization made by Leizeaga *et al*. [35] for samples of the oligotrophic Blanes Bay. After incubation, samples were fixed with 0.2-μm-filtered formaldehyde (3.7% v/v final concentration) overnight at 4°C. For each sample, a killed control was also incubated by fixing samples before HPG addition. Samples were then gently filtered through 0.6 and 0.2 μm pore-size polycarbonate filters for the eukaryotic and prokaryotic fractions, respectively, washed with 5 ml sterile milliQ water, and stored at −80°C until further processing. Before the click-reaction, cells were covered in agarose and samples for prokaryotes were also permeabilized with lysozyme and achromopeptidase as in Leizeaga *et al*. [35].

Cu(I)-catalyzed click-reaction was performed following the protocol described in Leizeaga *et al*. [35] using the CR110 azide fluorochrome (475/30 excitation and 527/54 BP emission). Same procedure was followed for the prokaryotic and eukaryotic samples using one-eighth filter sections. The click-reaction was performed into an Eppendorf tube containing the filter sections. The tube was covered with parafilm, without leaving air bubbles, and incubated 30 min at room temperature in the dark. After the click-reaction, the filters were washed, counterstained with 4′,6-diamidino-2-phenylindole (DAPI) [35], and analysed through epifluorescence microscopy.

### Identification and quantification of total and BONCAT-labelled eukaryotic cells

Eukaryotic cells were manually counted at a magnification of 1000× using an Olympus BX61 microscope. Cells with and without chlorophyll fluorescence (i.e. red fluorescence under blue light excitation) were classified as pigmented (which may include autotrophs and mixotrophs) and nonpigmented (heterotrophs), respectively. All counted cells were divided into two main size groups: small (≤5 μm) and large (>5 μm) eukaryotes. Small eukaryotes were further divided into ≤2, 3, 4, and 5 μm size categories. For large eukaryotes (>5 μm), two complete transects were examined from the centre to the edge of the filter, counting between 190 and 825 cells per sample, while for small eukaryotes, a minimum of 400 pigmented cells and 50 heterotrophic cells were counted. In total, eight eukaryotic groups were considered: pigmented (Pico P) and heterotrophic picoeukaryotes (Pico H) (2–3 μm), small pigmented (Nano P) and heterotrophic nanoeukaryotes (Nano H) (4–5 μm), pigmented (Dino P) and heterotrophic dinoflagellates (Dino H), and pennate (Diat P) and centric diatoms (Diat C). BONCAT-positive cells were detected by their green fluorescence under blue light, and for each eukaryotic group, positive and negative BONCAT cells were counted. Only cells with nucleus (visualized under UV excitation) were considered. Killed controls of all samples were checked to ensure that the HPG substrate was only incorporated into living cells, as no labelled cells were detected. The percentage of BONCAT-positive cells was calculated in relation to total counts (sum of all DAPI-stained cells).

### Image analysis for quantification of the HPG incorporation

The BONCAT-positive cell areas (μm^2^) were measured as a semiquantitative estimate of the HPG incorporation by the different groups. We used the area instead of fluorescence intensity, which has been shown to correlate well with the bulk heterotrophic activity [35], because it was not possible to set an exposure time that allowed the correct visualization of all groups due to the huge differences in cell size and BONCAT signal intensities. Four samples representing the largest differences in BONCAT-positive communities were selected for this analysis: 11 and 15 February both morning and afternoon samplings. For small eukaryotes (≤5 μm), abundances were converted to area assuming a circular shape and using the measured cell sizes (≤2, 3, 4, and 5 μm) and their abundances. However, within some of the largest cells, the BONCAT fluorescence signal was not equally distributed, but it rather showed the localization of the newly synthesized proteins. Hence, rather than the entire cell area, only the BONCAT-positive areas within diatoms and dinoflagellates (>5 μm) were measured manually using image analysis (see Supplementary Information for further details). Images were acquired using the motorized microscope ZEISS Axio Imager connected to a ZEISS camera (AxioCam MR3) and using the AxioVision 4.8 software. Images were taken at 400× magnification with the DAPI (UV excitation, 385 nm) and BONCAT defined channels (blue light excitation, 470 nm). BONCAT-positive areas of diatoms and dinoflagellates were manually measured from 150 images per sample with the ImageJ 1.53 software (https://imagej.net/ij/). The total BONCAT-positive area per ml associated to each group (diatoms and dinoflagellates) was calculated using the median area of cells and their cell abundances (Supplementary Information).

Total (DAPI-stained cells) and BONCAT-positive (DAPI-stained cells with BONCAT signal) prokaryotes were enumerated through automated image acquisition following the same procedure as for large eukaryotes, but using the 630× magnification. The percentage of BONCAT-positive cells and areas associated to prokaryotes were calculated with the ACMEtool2.0 (www.technobiology.ch) software analysing 35–75 images/sample.

### Statistical analyses

Redundancy analysis (RDA) was used to assess the BONCAT-positive community variation related to the measured environmental variables using the “rda” and “anova.cca” functions of the “vegan” R package [53]. Only noncollinear environmental variables were used for the model. Spearman’s correlations were used to assess the strength and direction of association between BONCAT-positive cell abundances and the environmental variables. Spearman’s Rho and *P-*values were calculated using the “cor” and “cor.test” functions in R. Student’s *t*-tests were used to compare means of morning versus afternoon measurements. Simple linear and multiple regression models were computed using the “lm” function in R.

## Results

### Visualization of BONCAT-positive eukaryotes

**Figure 1:**
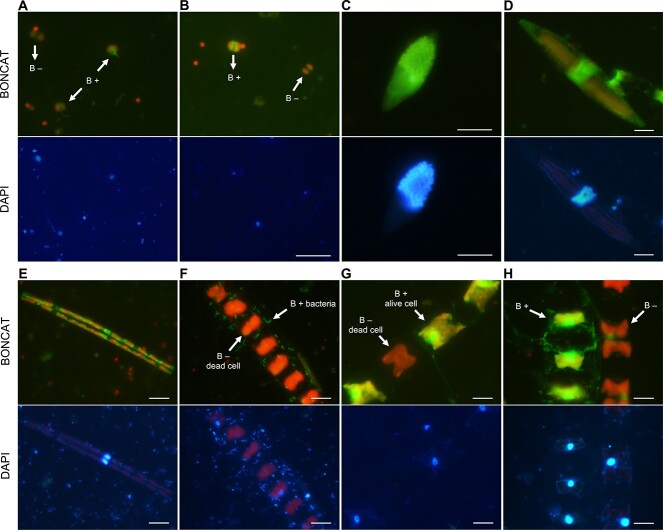
Microscopy images of BONCAT-positive cells; blue light (BONCAT) and UV light (DAPI) micrographs of eukaryotic cells; BONCAT fluorescence in bright green and chlorophyll fluorescence in red (more visible under blue light); B+ and B– indicate BONCAT-positive and negative cells, respectively; (A and B) pigmented pico- and nanoeukaryotes (≤5 μm); (C) B+ heterotrophic dinoflagellate; the DAPI image shows condensed chromosomes; (D and E) B+ pennate diatoms; (F) dead and B– chain of centric diatoms with associated B+ bacteria; (G) B+ chain of centric diatoms with one dead B– cell; (H) B+ and B– chains of alive centric diatoms. Scale bar indicates 10 μm for all images.

We observed a large diversity of eukaryotic cells labelled after HPG incorporation (hereafter, BONCAT-positive, B+), which could be clearly differentiated from the nonlabelled cells (B–) under epifluorescence microscopy (Fig. 1, Supplementary Fig. S1). Chloroplasts (red fluorescence) were visible in both B+ or B– cells, allowing to easily distinguish phototrophic from heterotrophic cells. Dead cells (cells with no visible nucleus in DAPI images) were never B+ (Fig. 1F–G), which, together with the absence of labelled cells in the killed controls, reinforces that the BONCAT fluorescence was specific to cells that were actively synthesizing proteins and using HPG as substrate and not from unspecific labelling or passive diffusion. BONCAT-negative cells in live samples might represent cells unable to take up or use the HPG (such as some groups like *Asterionellopsis* sp. that never appeared labelled, Supplementary Fig. S1) and also temporally inactive cells or resting stages produced by many eukaryotes, such as diatoms or dinoflagellates [54]. BONCAT fluorescence (in bright green) was found within many cell structures, yet it was observed with much more intensity around the nucleus (Fig. 1, Supplementary Fig. S1), and sometimes, in structures such as chloroplasts and flagella (Fig. 1, Supplementary Fig. S1). Some visually identifiable taxa were found to be always B+ (e.g. *Thalassiothrix* sp., Supplementary Fig. S1) and unicellular cyanobacteria (*Prochlorococcus* and *Synechococcus*), clearly visible under the microscope, were never found to be labelled.

### Eukaryotic community actively incorporating HPG

**Figure 2:**
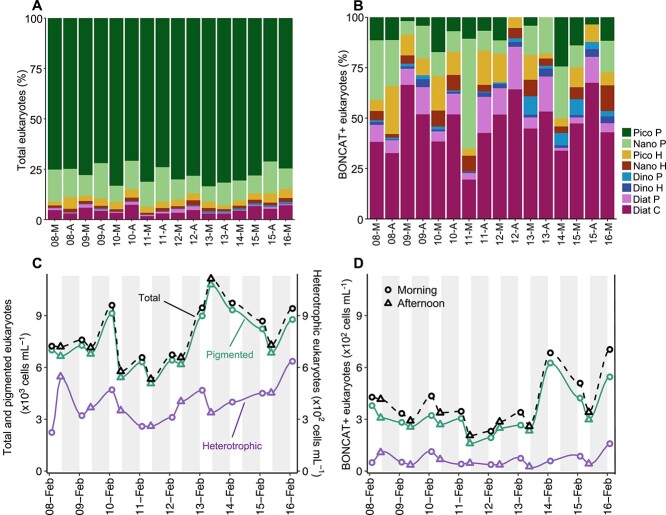
Total and BONCAT-positive eukaryotic community structure; relative contribution of the eukaryotic groups to the (A) total and (B) BONCAT-positive communities during the sampling period. *X*-axis labels indicate date in February 2021 and time of the day (M: morning; A: afternoon). Pico P: pigmented picoeukaryotes 2–3 μm, Nano P: pigmented nanoeukaryotes 4–5 μm, Pico H: heterotrophic picoeukaryotes 2–3 μm, Nano H: heterotrophic nanoeukaryotes 4–5 μm, Dino P: pigmented dinoflagellates, Dino H: heterotrophic dinoflagellates, Diat P: pennate diatoms, Diat C: centric diatoms. (C) Bulk (B+ and B−) and (D) BONCAT-positive cell abundances of pigmented (cells with chlorophyll signal, green line), heterotrophic (cells without chlorophyll signal, purple line), and total eukaryotes (pigmented + heterotrophic cells, dashed black line). *X*-axis labels indicate day-month and white-grey areas indicate day-night periods. Circles and triangles indicate morning and afternoon samplings, respectively. Note the different scale for eukaryotic groups in plot C.

The total and BONCAT-positive community structures were drastically different in terms of group’s relative abundances (Fig. 2). While the original community was largely dominated by pigmented picoeukaryotes (Pico P, 77.3 ± 4.2% cells, mean ± SD) followed by pigmented nanoeukaryotes (Nano P, 12.0 ± 3.3% cells) at all sampling times (Fig. 2A), the community of B+ eukaryotes was much more variable throughout the sampling period (Fig. 2B). In general, Diat C dominated the B+ cells community (47 ± 13% cells), followed by Nano P (16 ± 13% cells). Among all eukaryotic groups, dinoflagellates contributed the least both to the total (0.5 ± 0.3%) and the B+ community (4.5 ± 3.0%) (Fig. 2A and B). Altogether, the pigmented groups dominated both the total and BONCAT-positive communities throughout the study period (Fig. 2C and D), driving the observed overall eukaryotic abundance and HPG incorporation patterns.

**Figure 3:**
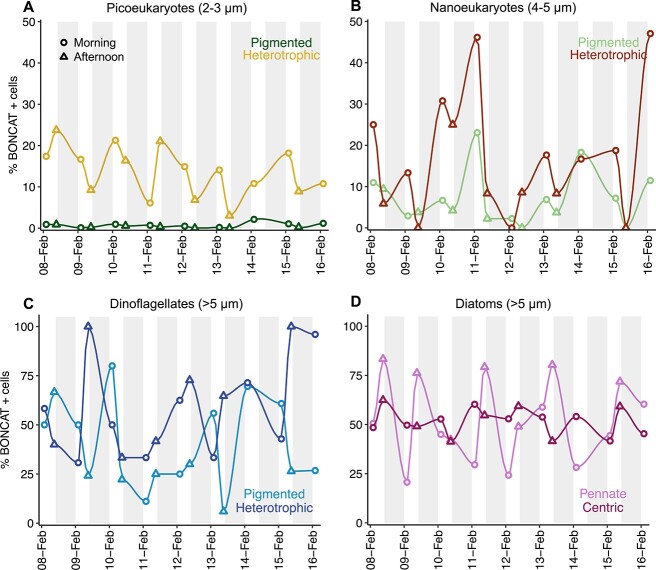
Temporal variability in the percentages of BONCAT-positive cells within the different eukaryotic groups: (A) pigmented and heterotrophic picoeukaryotes; (B) pigmented and heterotrophic nanoeukaryotes; (C) pigmented and heterotrophic dinoflagellates; and (D) pennate and centric diatoms. *X*-axis indicates day-month and white-grey areas indicate day-night periods. Circles and triangles indicate morning and afternoon samplings, respectively. Note the different scale for small (A and B) and large (C and D) eukaryotes.

Small eukaryotes (≤5 μm) had lower percentages of BONCAT-positive cells than large eukaryotes (>5 μm) (Fig. 3) and, in general, small heterotrophic groups showed higher osmotrophic activity than their phototrophic counterparts, ranging between 3-24% and 0-47% of B+ cells in heterotrophic pico- and nanoeukaryotes, respectively, and 0-2% and 0-23% of B+ cells in phototrophic pico- and nanoeukaryotes, respectively (Fig. 3A and B). The percentage of B+ dinoflagellates was highly variable throughout the study, varying between 6-80% and 31-100% of B+ cells in pigmented and heterotrophic dinoflagellates, respectively (Fig. 3C). 21–83% of pennate diatoms were B+ throughout the study and showed a marked morning–afternoon periodicity (Fig. 3D), whereas centric diatoms were less variable in BONCAT incorporation than the other large eukaryotic groups, displaying between 41 and 62% of B+ cells (Fig. 3D).

Based on the 18S rRNA gene sequencing (Supplementary Fig. S2), the pico-sized fraction was dominated by the photosynthetic groups Mamiellophyceae, Prymnesiophyceae, and Pelagophyceae. The most important heterotrophic taxa were marine alveolates (MALV I–III) and several MAST clades. Other important groups found in this fraction, like ciliates and diatoms, were detected likely due to cell breakage during filtration. The large size fraction (3–20 μm) was predominantly constituted by Diatomea and Dinoflagellata taxa supporting our microscopy observations and, although the largest cells (>20 μm) were theoretically excluded from this size fraction, some of the most abundant genera detected included the centric diatoms *Chaetoceros*, *Thalassiosira*, *Lauderia*, and *Asterionellopsis* and the pennate diatoms *Pseudo-nitzschia* and *Haslea*, which agrees well with the taxa identified by microscopy (Supplementary Fig. S1). *Gymnodinium*, *Ptychodiscus*, and *Karenia* were the dominant phototrophic and mixotrophic dinoflagellates, and *Gyrodinium* and *Warnowia* dominated the sequences within heterotrophic dinoflagellates (Supplementary Fig. S2).

### Short-term variability in eukaryotic HPG incorporation and its drivers

**Figure 4:**
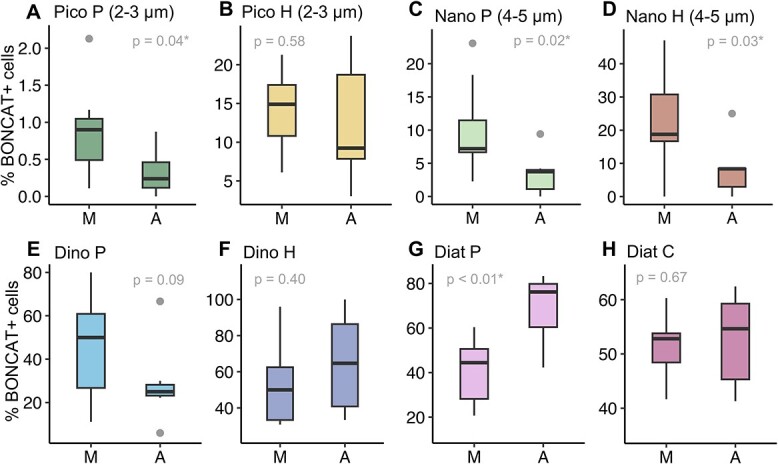
Morning versus afternoon eukaryotic HPG incorporation. Variation in the percentages of BONCAT-positive cells between morning (M) and afternoon (A) samplings for the different eukaryotic groups: (A) pigmented (Pico P) and (B) heterotrophic picoeukaryotes (Pico H), (C) pigmented (Nano P) and (D) heterotrophic nanoeukaryotes (Nano H), (E) pigmented (Dino P) and (F) heterotrophic dinoflagellates (Dino H), (G) pennate (Diat P) and (H) centric diatoms (Diat C) Data beyond the end of the whiskers (outliers) are represented as grey dots in the boxplots; significance (*P-*value, Student’s *t*-test) of morning versus afternoon variation is indicated for each boxplot; significant differences (*P* < 0.05 ) are indicated by an asterisk.

Despite the observed temporal variability in the percentage of B+ cells within most groups (Fig. 3), only four groups showed significant variation in their percentages of B+ cells between the morning and afternoon sampling times (Fig. 4). Pigmented picoeukaryotes (Pico P) and both groups of nanoeukaryotes (Nano P and Nano H) showed significantly higher percentages of B+ cells in the morning than in the afternoon (Fig. 4A, C, and D). Contrarily, pennate diatoms showed significantly higher proportions of B+ cells in the afternoon than in the morning (Fig. 4G). Heterotrophic picoeukaryotes (Pico H), dinoflagellates, and centric diatoms did not show a significant diel periodicity or a clear temporal pattern in HPG incorporation (Figs 3 and 4). Although total and B+ cell abundances were positively correlated in all cases (Supplementary Fig. S3), none of the studied groups showed significant differences in the total cell abundances between morning and afternoon samplings (Supplementary Fig. S4).

**Figure 5:**
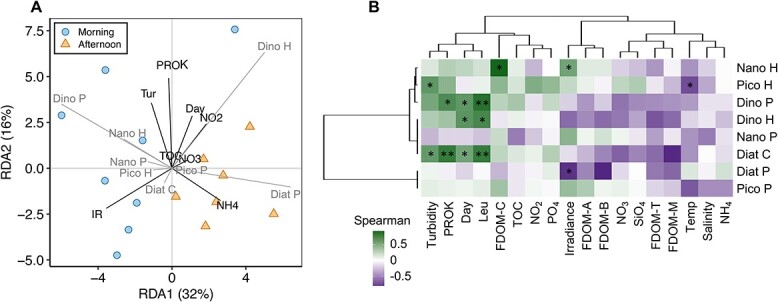
Environmental drivers of the eukaryotic BONCAT-positive community. (A) RDA of the eukaryotic BONCAT+ communities and environmental-biotic variables. The percentage of variance explained is shown for each axis; the direction and length of vectors represent the direction of increase and strength of relative correlations with the continuous variables. Only noncollinear variables were included in the model. Circles and triangles indicate morning and afternoon samplings, respectively. Results of the model are shown in Supplementary Table S1. (B) Clustered heatmap of Spearman correlations between BONCAT+ cell abundances of the different groups and environmental-biotic variables. Please note that FDOM measurements were only available for morning samplings (*n* = 9), whereas the rest were available for all time points (*n* = 16). Significant correlations are indicated by asterisks (^*^  *P* < 0.05, ^*^^*^  *P* < 0.01). Tur: turbidity; PROK: prokaryotic abundance; Day: day of sampling; Leu: leucine incorporation rates; IR: irradiance level; Temp: temperature.

Regarding the factors explaining changes in the eukaryotic incorporation of HPG, we found that time of the day (i.e. morning vs. afternoon) (Fig. 5A and Supplementary Table S1) was the variable showing the largest influence on the overall structuring of the B+ community, explaining 23% of the B+ community variability. The abundance of B+ cells of heterotrophic nanoeukaryotes and pennate diatoms, which were groups showing clear morning–afternoon changes in activity (Fig. 4), were positively and negatively correlated with irradiance, respectively (Fig. 5B), in agreement with the irradiance level differences between the two sampling times (Supplementary Fig. S5).

Other measured variables also showed significant correlations with the B+ cell abundances of different groups. For example, the abundance of B+ heterotrophic picoeukaryotes was positively correlated with turbidity (Fig. 5B). In general, TOC or the quality of DOM did not explain changes in the B+ abundances of any group except heterotrophic nanoeukaryotes, which were positively correlated to FDOM-C (Fig. 5B), although FDOM was only measured once a day in the morning. Centric diatoms and dinoflagellates (Dino P and Dino H) were significantly and positively influenced by the day of sampling (Fig. 5B), which indicates a gradual increase in their B+ cell abundances over the sampling period.

### Eukaryotic versus prokaryotic HPG incorporation

**Figure 6:**
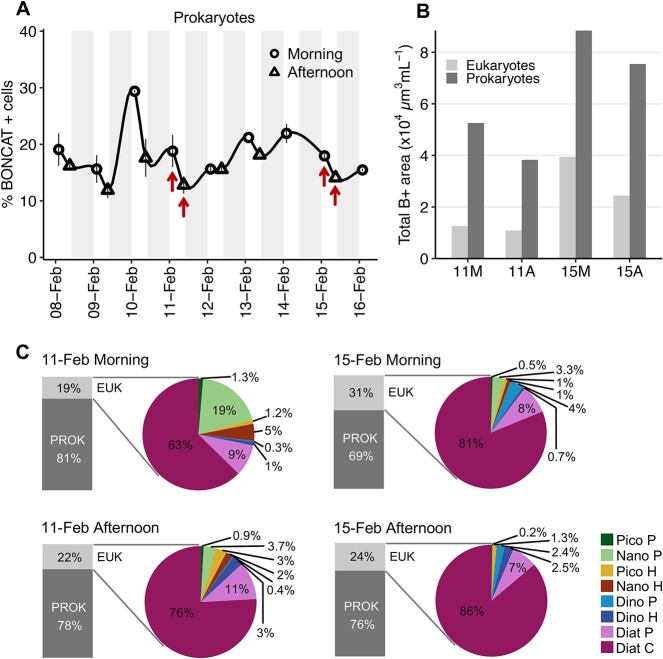
Relative contribution of eukaryotes versus prokaryotes to total HPG incorporation. (A) Temporal variability in the percentages of BONCAT-positive prokaryotes with respect to total prokaryotic cells. *X*-axis labels indicate sampling day (day-month) and white-grey areas indicate day-night periods. Circles and triangles indicate morning and afternoon samplings, respectively; red arrows indicate the four samples where the prokaryotic and eukaryotic BONCAT signals were compared. (B) Total BONCAT-positive areas (in μm^3^ ml^−1^) associated to eukaryotic and prokaryotic communities in the four selected samples. (C) Relative contribution of prokaryotes (PROK) and eukaryotes (EUK) to total community HPG incorporation in the four selected sampling times, indicating the relative contribution of the different eukaryotic groups to total eukaryotic community HPG incorporation.

To compare the contribution to HPG incorporation of the different eukaryotic groups with respect to prokaryotes, we selected four samples (11 and 15 February morning and afternoon) representative of some of the most different samples in terms B+ community composition: 11 February, characterized by minimum total and B+ cell abundances, both eukaryotic and prokaryotic, and by the smallest contribution of centric diatoms and the largest of photosynthetic nanoeukaryotes (Nano P) to the eukaryotic B+ community. Conversely, 15 February had a higher abundance and contribution of B+ centric diatoms cells (Fig. 2B). Similar percentages of B+ prokaryotes were detected in both days (Fig. 6A).

We estimated that 19–31% of the total (prokaryotic + eukaryotic) BONCAT signal (inferred as the BONCAT+ area) was channelled through eukaryotes (Fig. 6B and C). The sample with the lowest percentage of eukaryotic HPG incorporation (19%) was that of the morning of 11 February, characterized by the lowest abundance of B+ centric diatoms and the highest abundance of B+ pigmented picoeukaryotes. Conversely, the highest eukaryotic contribution to total HPG uptake (31%) was observed in the morning of 15 February due to the higher abundances of B+ centric diatoms and pigmented dinoflagellates in spite of the larger B+ area associated to prokaryotes (Fig. 6B).

Among the eukaryotic groups, centric diatoms were by far the dominant group incorporating HPG, comprising 63–86% of the total eukaryotic BONCAT signal (Fig. 6C). The largest B+ cells observed in all samples belonged to centric diatoms (likely *Chaetoceros*, *Lauderia*, and *Thalassiosira spp.*), most of them forming long cell chains (Supplementary Fig. S1). Pennate diatoms, likely *Pseudo-nitzschia* spp., among others, were also important HPG consumers, representing 7–11% of the total eukaryotic BONCAT signal. Photosynthetic nanoeukaryotes contributed considerably (19%) to the community of the morning of 11 February due to their high B+ cell abundance (Fig. 2B). Dinoflagellates represented low contributions to eukaryotic HPG incorporation since, in general, most of them were smaller (5–10 μm of diameter) and much less abundant than diatoms.

**Figure 7:**
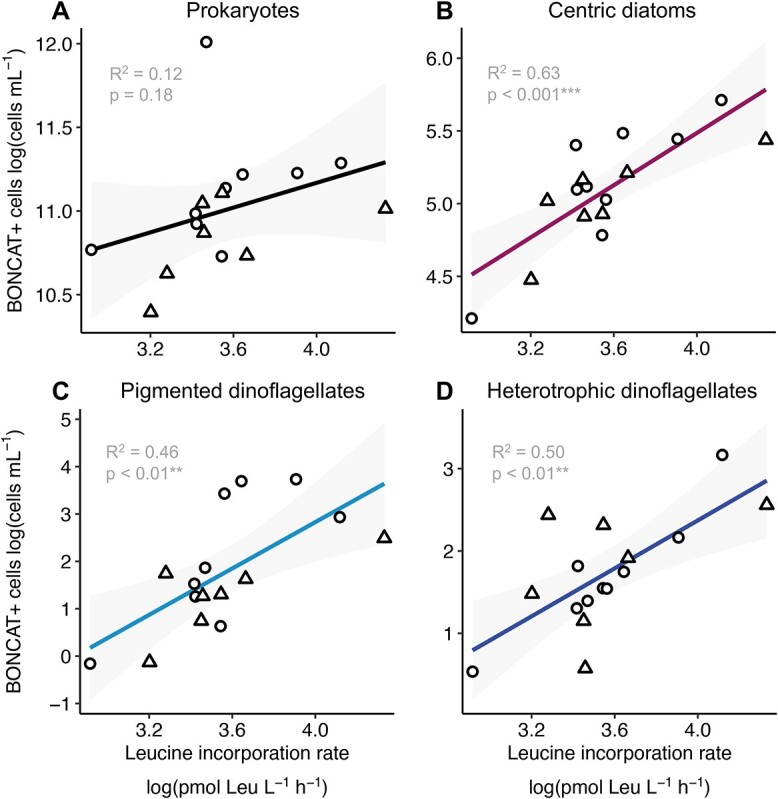
Relationships between BONCAT-positive cells and bulk ^3^H-leucine incorporation rates. Linear regressions between the BONCAT+ cell abundances of (A) prokaryotes, (B) centric diatoms, (C) pigmented dinoflagellates, and (D) heterotrophic dinoflagellates (log cells ml^−1^) with leucine incorporation rates (log pmol Leu l^−1^ h^−1^). The *R*^2^ and *P-*values are indicated for each linear model, asterisks indicate the significance of the relationship. Shaded grey areas indicate the 95% confidence interval of the regression slope. Circles and triangles indicate morning and afternoon samplings, respectively.

Interestingly, the bulk ^3^H-leucine incorporation rates were positively and significantly correlated to the abundance of B+ centric diatoms and pigmented and heterotrophic dinoflagellates (Figs 5B and 7B–D), whereas the correlation with the abundance of B+ prokaryotes (Fig. 7A), the other eukaryotic groups, and the total community cell abundance (prokaryotes + eukaryotes) (Supplementary Fig. S6) was not statistically significant (*P* >0.1 ). The abundance of BONCAT-positive centric diatoms explained up to 63% of the variance in leucine incorporation rates, followed by heterotrophic dinoflagellates that explained around 7%. Only 0.5% of leucine incorporation variability was explained by the abundance of BONCAT+ prokaryotes (multiple *R*^2^ of the regression model = 0.83) (Supplementary Table S2).

## Discussion

Here, we show the potential of BONCAT for readily assessing and quantifying the eukaryotic osmotrophic incorporation of a methionine analogue in natural communities, which allows identifying groups of microorganisms contributing to its uptake. Our results suggest a widespread capacity to incorporate HPG among different planktonic eukaryotes, which potentially compete with prokaryotes for its use, and highlight that the short-term variability in HPG consumption depends both on community taxonomic composition and on single-cell changes in activity within and across eukaryotic groups.

### BONCAT: a useful tool to readily assess microbial osmotrophic HPG incorporation

The application of BONCAT allowed identifying a large diversity of phototrophic and heterotrophic eukaryotes that had actively incorporated the methionine analogue into proteins. The fluorescent signal was generally located around the nucleus, where most cytoplasmic ribosomes are found in eukaryotic cells and where protein synthesis occurs [55], as well as in chloroplasts and flagella (Fig. 1 and Supplementary Fig. S1), which reinforces that the observed fluorescence comes from the incorporated amino acid into cellular structures. BONCAT-positive bacteria attached to algae could easily be distinguished from both BONCAT-positive or -negative eukaryotic cells, supporting that the signal was not attributed to any surface-attached bacteria. This represents an advantage with respect to microautoradiography, where the label around substrate-incorporating cells can be wider and it may overlap with the signal of any surface-associated prokaryote [21].

BONCAT has been used to label newly synthesized proteins in a wide range of cell types, such as mammal neurons [56], insect [57] or plant [58] tissues. The application of BONCAT to planktonic eukaryotes has been limited to a few cultured species (*E. huxleyi*, *C. burkardae*, *Ostreococcus* sp., and *M. pusilla*), showing efficient incorporation of the two different methionine analogues L-azidohomoalanin (AHA) [44] and HPG [38, 43]. The latter two studies also proved that the growth dynamics of the cultured photosynthetic eukaryotes were not altered when using final HPG concentrations of up to 100 μM, suggesting that the concentration used here (2 μM during 2 h of incubation) did not alter the normal growth dynamics of the community. This incubation time and the HPG concentration were chosen based on a previous optimization of the method with bacterial samples from the Blanes Bay, in which using 1–2 μM and 2–3 h of incubation was recommended [35], and we wanted to explore the capacity of eukaryotes to take up HPG under the same conditions used for bacterial samples. This HPG concentration was higher than the methionine concentration found naturally in the study site (ca. 40 nM in May [59]), but it was needed because the affinity for HPG is 10 times lower than for methionine [39], and concentrations <500 nM failed to detect a large fraction of protein-synthesizing cells [35]. Previous work has also shown that increasing the HPG concentration from 20 nM to 2 μM results in a small increase in the BONCAT+ cells detected [39], indicating that this high substrate concentration does not result in the induction of BONCAT+ cells. Finally, although it is unknown whether some of the incorporated HPG can be released back into the medium during incubations, the proportion of active bacterial planktonic cells seems to behave linearly with the incubation time for at least 4 h [35] (Supplementary Fig. S7). This suggests that little crossfeeding could occur during our 2-h incubations and thus our approach should not be overestimating the actual HPG uptake capacities of the studied groups.

### Prevalent HPG incorporation among large eukaryotic phytoplankton

Large eukaryotes (diatoms and dinoflagellates) exhibited higher and more variable percentages of BONCAT-positive cells than small eukaryotes (≤5 μm), suggesting that they are active consumers of HPG. This agrees with previous microautoradiography-based evidences of incorporation of other amino acids, such as leucine by diatoms and dinoflagellates from different marine regions [21], and supports that the uptake of exogenous amino acids may supplement the phototrophic and/or heterotrophic growth in these groups [23]. Actually, many diatom species are facultative mixotrophs, and some are even able to grow in darkness using organic carbon sources [60]. The 18S rRNA sequence data indicated the presence of genera such as *Chaetoceros*, *Thalassiosira*, *Pseudo-nitzschia*, and *Prorocentrum*, all which have been previously shown to take up organic substrates [10, 21, 29, 30, 61]. The uptake of HPG measured by BONCAT may hence represent a quick way to address the potential osmotrophic activity of dominant phytoplankton groups and to better quantify the relevance of this process in nature.

Methionine is not only used in protein synthesis, but it can also be used as a precursor of several important sulphur-containing metabolites, such as dimethylsulfoniopropionate (DMSP) [62] or S-adenosylmethionine [63]. Gage *et al*. [64] estimated that ~60% of radiolabelled methionine added to a culture of the marine algae *Ulva intestinalis* was incorporated into proteins after 2 h of incubation, while the rest of it was mainly converted to DMSP or remained free. Given that BONCAT only detects the methionine analogue (HPG) when incorporated into proteins [33], our results likely underestimate the total eukaryotic uptake of methionine.

Besides osmotrophy, many eukaryotic phytoplankton groups are also capable of phagotrophy (the engulfment of organic particles or prey) [5, 65], and hence the BONCAT signal might also represent, in some cases, incorporation of the substrate from phagotrophy on bacteria [5, 17, 66, 67]. While we did not directly observe any apparent BONCAT-positive ingested bacteria in any of the eukaryotic organisms, the transfer of HPG-labelled proteins from cultured *Escherichia coli* and *E. huxleyi* hosts to their viruses during lytic infection has been reported [43], supporting the plausible transfer of HPG-labelled proteins from preys to predators. In any case, the fact that diatoms dominated the eukaryotic substrate incorporation, both in terms of BONCAT-positive cell abundance and area, supports that osmotrophy was the main pathway of HPG incorporation in the studied communities, as diatoms capable of phagotrophy are not known to date [5, 16]. BONCAT may thus help complement the current omics efforts to better constrain the role of mixotrophy in organic matter flows in the ocean [16, 68], and future efforts to synthetize BONCAT surrogate substrates other than HPG and AHA would widen its potential to explore eukaryotic osmotrophy [36].

### Short-term variations in eukaryotic organic substrate incorporation

The eukaryotic community structure was consistent with that previously reported for this area and time of year [69, 70]. Some changes in TOC, nutrient concentration, and DOM quality were observed during the study period, yet these factors were not clearly related to the observed osmotrophic activity variations. Nonetheless, several groups did show recurrent morning–afternoon changes in the incorporation of HPG. Light is a major factor determining the diel periodicity of many metabolic pathways, including amino acid uptake [21, 24] and protein synthesis [71], and affects other processes such as bacterivory [72, 73]. However, studies with cultures and natural communities showed a variable influence of light on uptake patterns depending on the species and the compound [9, 21, 22, 28], complicating our understanding of osmotrophy regulation in natural communities. For example, pennate diatoms displayed a higher osmotrophic activity in the afternoon than in the morning and a negative correlation with irradiance, which is in accordance with previous results showing ^3^H-leucine uptake by *Pseudo-nitzschia* and *Navicula* being negatively affected by solar radiation [21]. Conversely, the opposite pattern shown by small eukaryotes may had been influenced by phagotrophy, which was shown to peak at night at the study site [73].

We focused on the morning versus afternoon comparison because we were interested in short-term changes in osmotrophy during the daylight hours where photosynthesis also takes place, but it is likely that HPG incorporation also changes between day and night and on a seasonal basis, as reported for other organic substrates [10, 22, 25]. Although incubations with HPG must be performed in the dark [35], the morning–afternoon variations observed for some groups could point to a role of light on regulating HPG incorporation (as morning/afternoon communities had been exposed to different light levels prior to collection) or to an endogenous circadian regulation of HPG uptake, as observed for the amino acid uptake in *Synechococcus* [22]. Also, different taxonomic groups of planktonic eukaryotes have shown different diel transcriptional patterns of protein-encoding genes [74], which could explain the distinct morning versus afternoon protein synthesis patterns observed in our study. Dinoflagellates and centric diatoms did not show clear morning–afternoon variations as a whole, but since we could not identify them at the species level, it is possible that specific activity trends remained masked. The combination of BONCAT with Catalyzed Reporter Deposition Fluorescent *in situ* Hybridization [34] offers a promising way to accurately identify specific eukaryotic groups at a higher taxonomic resolution and link them to activity.

### Eukaryotic versus prokaryotic substrate incorporation

When prokaryotes were also considered, we estimated that eukaryotes accounted for a notable share (19–31%) of the bulk HPG incorporation (Fig. 6), with centric diatoms accounting for most of the eukaryotic BONCAT signal. We used BONCAT-positive areas as a semiquantitative measure of HPG incorporation, given that the BONCAT signal intensity, previously shown to be proportional to protein synthesis rates in prokaryotes [35, 39], could not be assessed here due to its high variability among cell sizes (from <2 to >100 μm). However, given the high signal intensity of centric diatoms, and considering that we are likely overestimating the BONCAT-positive area of small eukaryotic cells since we considered their entire cell area as BONCAT-positive, our approach probably underestimates the actual contribution of centric diatoms to total HPG incorporation.

Remarkably, the abundance of BONCAT+ centric diatoms was positively correlated with the bulk ^3^H-leucine incorporation rates, accounting for most (63%) of its variability during the study period. ^3^H-leucine incorporation rates are widely used as a proxy of prokaryotic heterotrophic production [47, 48], and they have been previously found to correlate well with the BONCAT signal intensity in prokaryotes [35]. In view of our results and previous reports of diatoms strongly labelled for ^3^H-leucine at the study site [75], the fact that several of the phytoplankton groups showed stronger correlations with bulk ^3^H-leucine uptake than BONCAT+ prokaryotes suggests that osmotrophic phytoplankton, and particularly centric diatoms, may be responsible for a significant share of ^3^H-leucine uptake. This warns that estimates of prokaryotic production based on amino acid incorporation might be largely impacted by the eukaryotic osmotrophic activity, at least in surface microbial communities with high abundances of mixotrophic phytoplankton.

Finally, the changes in phytoplankton assemblages at the study site towards fewer diatoms, more cyanobacteria, and increases in bacterial abundance and production in summer [76, 77] could result in a decreased eukaryotic HPG consumption in seasons other than winter. However, repeated observations of BONCAT+ phytoplankton cells in samples from other regions (Supplementary Fig. S8), such as the North Atlantic (Gómez-Letona *et al*., unpublished work) and the eutrophic Mar Menor coastal lagoon in the Mediterranean (Mena *et al*., unpublished work), highlight the ubiquity of this behaviour and support the broad applicability of the BONCAT method.

Taken together, our results reveal that the osmotrophic uptake and incorporation of HPG are widely distributed among different taxonomic and functional eukaryotic groups and that the importance of this process varies significantly at short temporal scales (hours, days) depending on group-specific variation in abundance and activity. Such a complex regulation of the uptake of a single organic substrate suggests that understanding the eukaryotic use of dissolved organic compounds is likely extremely difficult, but it highlights an important role of phytoplankton osmotrophy in carbon flow dynamics [78]. Diatoms were found to be the main channellers of HPG and appeared to determine temporal variations in bulk ^3^H-leucine incorporation rates; so, given their widespread distribution and ecological relevance in the global ocean [79], diatom osmotrophy may be key for understanding element cycles, carbon sequestration, and food web dynamics in the ocean.

## Supplementary Material

Supplementary_Mena_et_al_ISMEComm_ycae004

## Data Availability

Raw sequences are publicly available at the European Nucleotide Archive (https://www.ebi.ac.uk/ena) under the accession number PRJEB63614. The datasets generated for this study are available on request to the corresponding authors.
