# Supplementary material for: High amino acid osmotrophic incorporation by marine eukaryotic phytoplankton revealed by click chemistry
Source: ISME Commun. 2024 Jan 17;4(1):ycae004. doi: 10.1093/ismeco/ycae004 (PMC10902890; doi:10.1093/ismeco/ycae004)
Supplement: Supplementary_Mena_et_al_ISMEComm_ycae004 [file supplementary_mena_et_al_ismecomm_ycae004.pdf]

High amino acid osmotrophic incorporation by marine eukaryotic phytoplankton revealed by click-chemistry

Catalina Mena[1]*, Ona Deulofeu-Capo1, Irene Forn[1], Júlia Dordal-Soriano[1], Yulieth A. Mantilla-Arias[1], Iván P. Samos[1], Marta Sebastián[1], Clara Cardelús[1], Ramon Massana[1], Cristina Romera-Castillo[1], Rebeca Mallenco-Fornies[1], Josep M. Gasol[1] and Clara Ruiz-González[1]*

[1]Department of Marine Biology and Oceanography, Institut de Ciències del Mar (ICM-CSIC), 08003, Barcelona, Spain.

*Corresponding authors: cmena@icm.csic.es, clararg@icm.csic.es

SUPPLEMENTARY MATERIAL

Supplementary Information
BONCAT-positive area calculation 2

Supplementary Figures
Fig. S1. Microscopy images 6
Fig. S2. Eukaryotic community composition 11
Fig. S3. Total and BONCAT-positive abundance correlations 12
Fig. S4. Morning *vs*. afternoon abundances 13
Fig. S5. Environmental data 14
Fig. S6. Correlation between B+ cell abundances and leucine incorporation rates . 16
Fig. S7. BONCAT time course experiment with bacteria 17
Fig. S8. Microscopy images from other marine sites. 18

Supplementary Tables
Table S1. RDA eukaryotic BONCAT-positive community 19
Table S2. Multiple regression model BONCAT-Leucine. 19

SUPPLEMENTARY INFORMATION

BONCAT-positive area calculation for large eukaryotes:

BONCAT-positive areas of large eukaryotic groups (pigmented and heterotrophic (non-pigmented) dinoflagellates and pennate and centric diatoms) were measured manually through image analysis following the methodology described below:

1. We first delimited the areas of BONCAT fluorescence within cells, as shown in Fig. A, and classified them within the four eukaryotic groups. Since BONCAT is not equally distributed within cells (green fluorescence shows an approximate location of newly synthesized proteins using the HPG substrate), only BONCAT-positive areas were measured (Fig. A). Images were acquired using the motorized microscope ZEISS Axio Imager connected to a ZEISS camera (AxioCam MR3) and using the AxioVision 4.8 software. Images were taken at 400× magnification using the DAPI (UV excitation, 385 nm) and BONCAT (blue light excitation, 470 nm) defined channels. BONCAT-positive areas from 150 images per sample were then manually measured with the ImageJ 1.53 software.

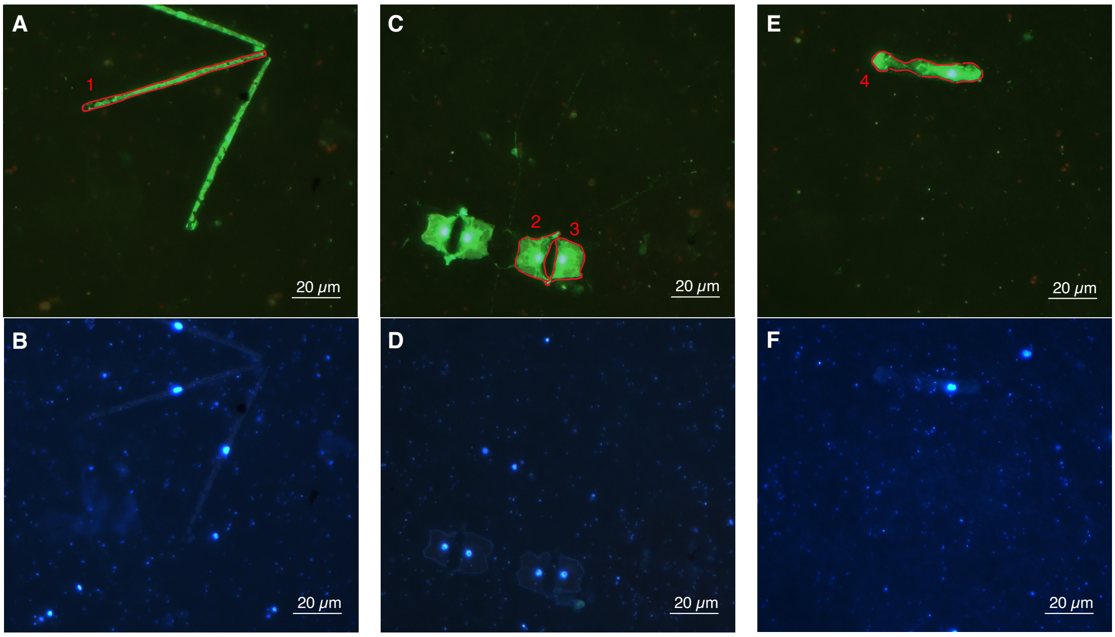

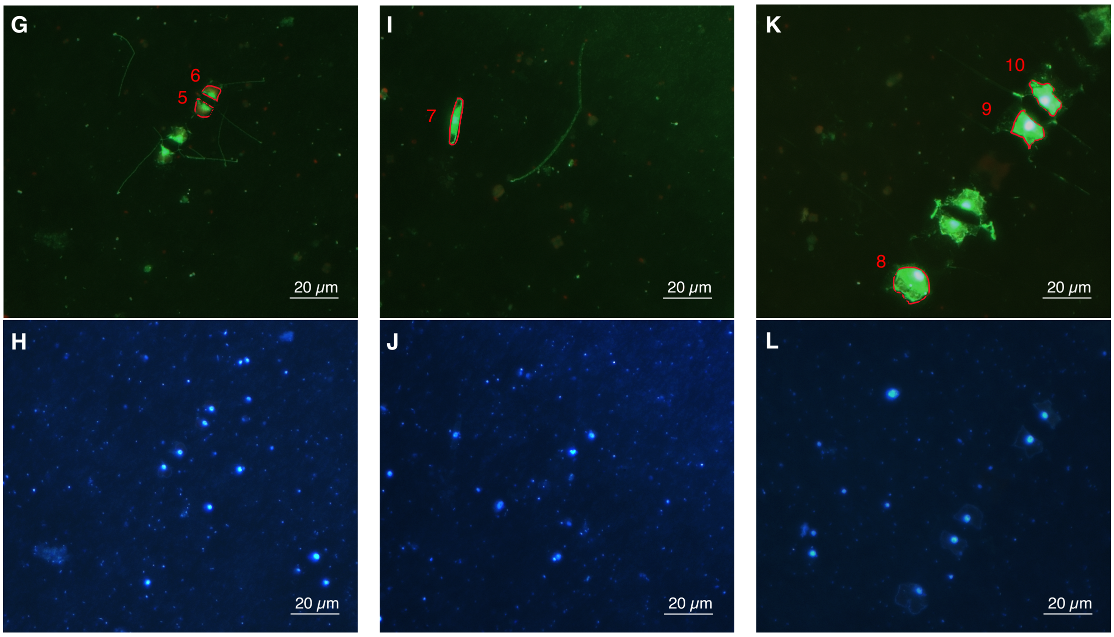

Figure A. Images of eukaryotic cells taken using blue light excitation (BONCAT-positive cells emit green fluorescence, **A,C,E,G,I,K**) and UV excitation (DAPI, nucleic acids in bright blue, **B,D,F,H,J,L**). In red are delimited the BONCAT-positive areas of some of the BONCAT-positive cells as examples, this procedure was used for all large eukaryotic BONCAT-positive cells. A-B and I-J images are examples of pennate diatoms and C-H and K-L of centric diatoms.

Figure B shows an example of the distribution of the measured BONCAT-positive areas for the four eukaryotic groups of one of the samples analysed (February 11th, afternoon). Note that although most cells fell within smaller sizes, there were also a few very large cells within each group, and hence the mean or median area values were not representative of the mean or median size of the largest and most active cells.

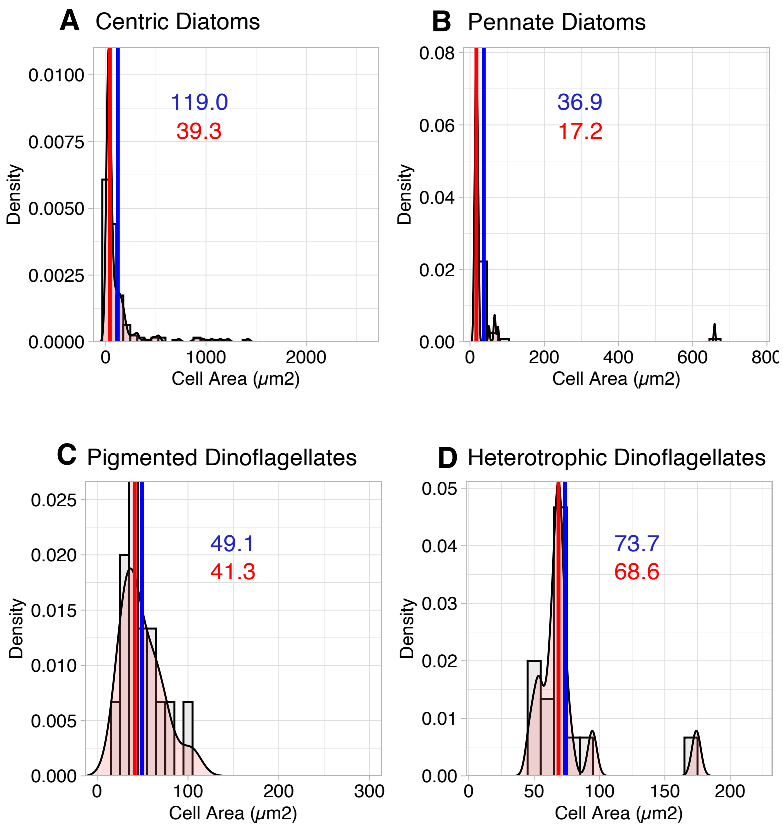

Figure B. Distribution of the BONCAT-positive cell areas of (**A**) centric diatoms, (**B**) pennate diatoms, and (**C**) pigmented and (**D**) heterotrophic dinoflagellates measured in sample 11 Feb afternoon. Red and blue vertical lines indicate mean and median, respectively, and their associated values are also indicated in each plot in the corresponding color.

2. To convert the measurements of BONCAT-positive areas (μm^2) to area mL^{-1}, we considered four size-ranges in each of the targeted groups in order to avoid underestimating larger cells.

2.1. BONCAT-positive area data were divided into four ranges (Fig. C), and the proportion of cells within each size-range was calculated for each sample (Table A).

2.2. The cell abundance for each size-range was calculated using the BONCAT-positive cell abundances from microscopy counts and the % of cells within each size-range (Table A).

2.3. Then, the median of the BONCAT-positive areas was calculated for each range and multiplied by the corresponding cell abundance (cells mL^{-1}), in order to obtain the BONCAT-positive area associated to each size-range in μm^2 mL^{-1}.

2.4. Finally, the total BONCAT-positive area associated to a given group was estimated as the sum of the area mL^{-1} associated to the cells within each of the four size-ranges.

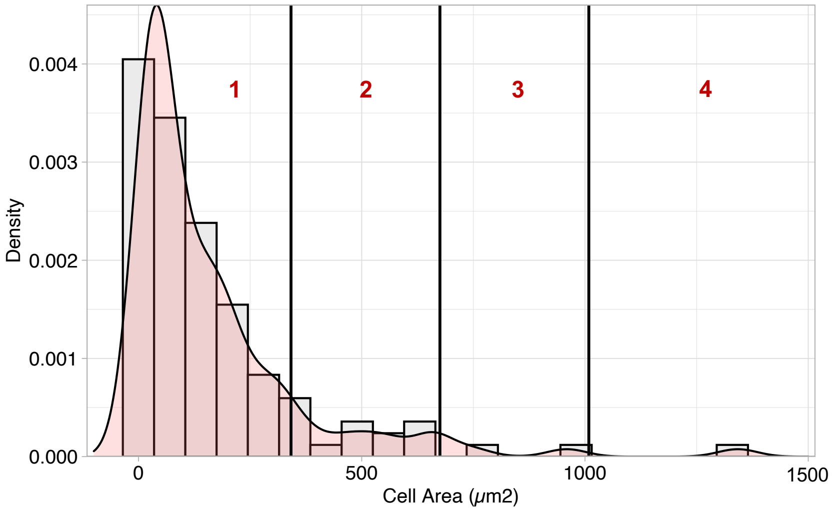

Figure C. Histogram of areas measured for centric diatoms in the morning sample of Feb 11, and their division in the four size-ranges numbered from 1 to 4. Detailed calculations are shown in Table A.

Table A. Example of calculations for the morning sample of Feb 11, depicted in Fig. C above. Total abundance indicates the total centric diatom density calculated from microscopy counts for this sample.

	Total abundance	67.41 cells mL^{-1}			
	Size-ranges	1	2	3	4
2.1	Area range (μm^2)	8.1 – 341.6	341.6 – 675.1	675.1 – 1008.6	1008.6 – 1342.1
	% of cells within each range	88.3	9.2	1.7	0.8
2.2	Abundance in each size-range (cells mL^{-1})	59.52	6.20	1.14	0.54
2.3	Median BONCAT-positive cell area (μm^2)	50.9	514.4	853.2	1342.1
	BONCAT-positive area (μm^2 mL^{-1})	3029.57	3189.28	972.65	724.73
2.4	Total BONCAT-positive area	7916.23 μm^2 mL^{-1}			

SUPPLEMENTARY FIGURES

FIGURE S1

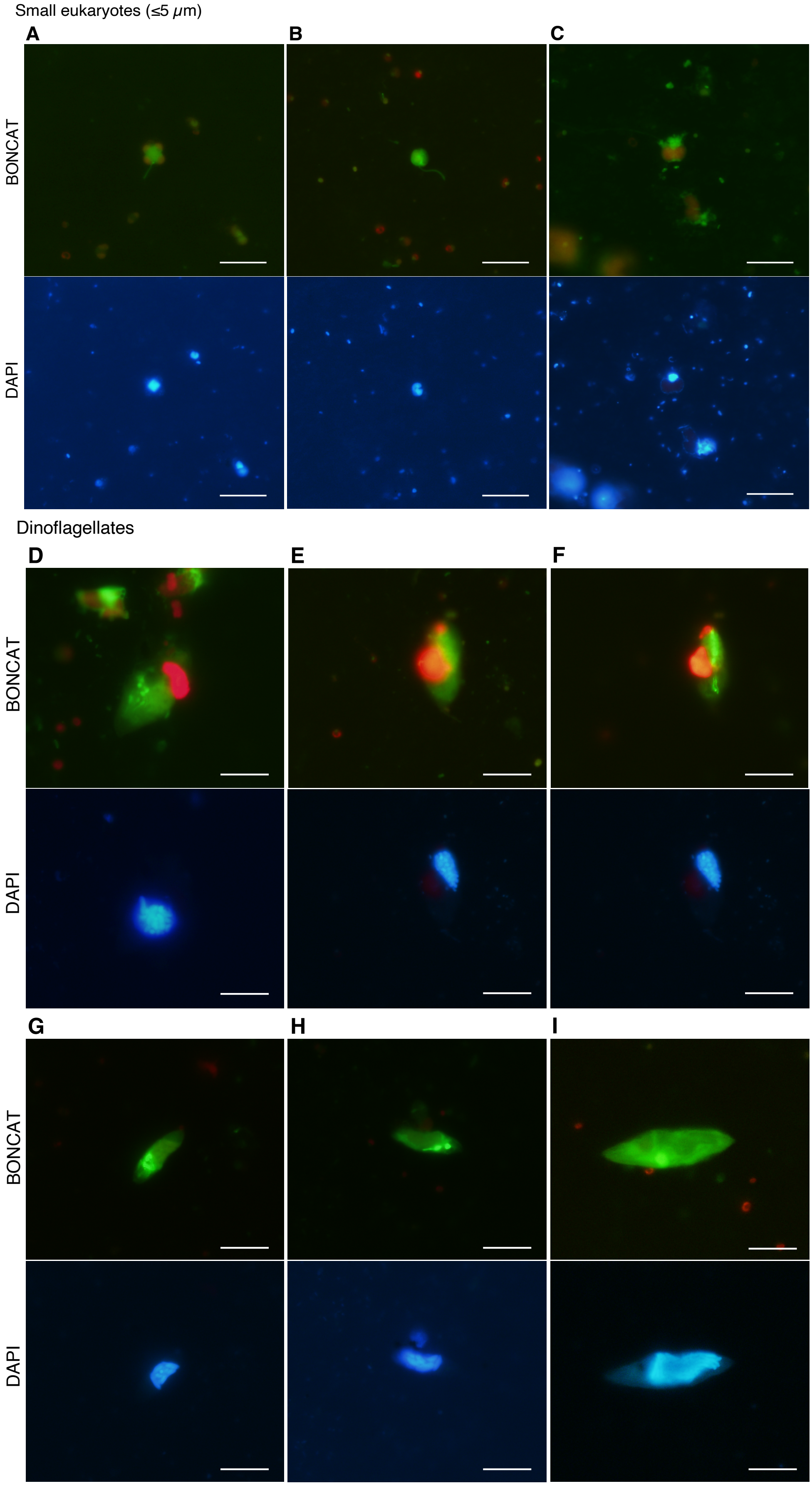

Pennate diatoms

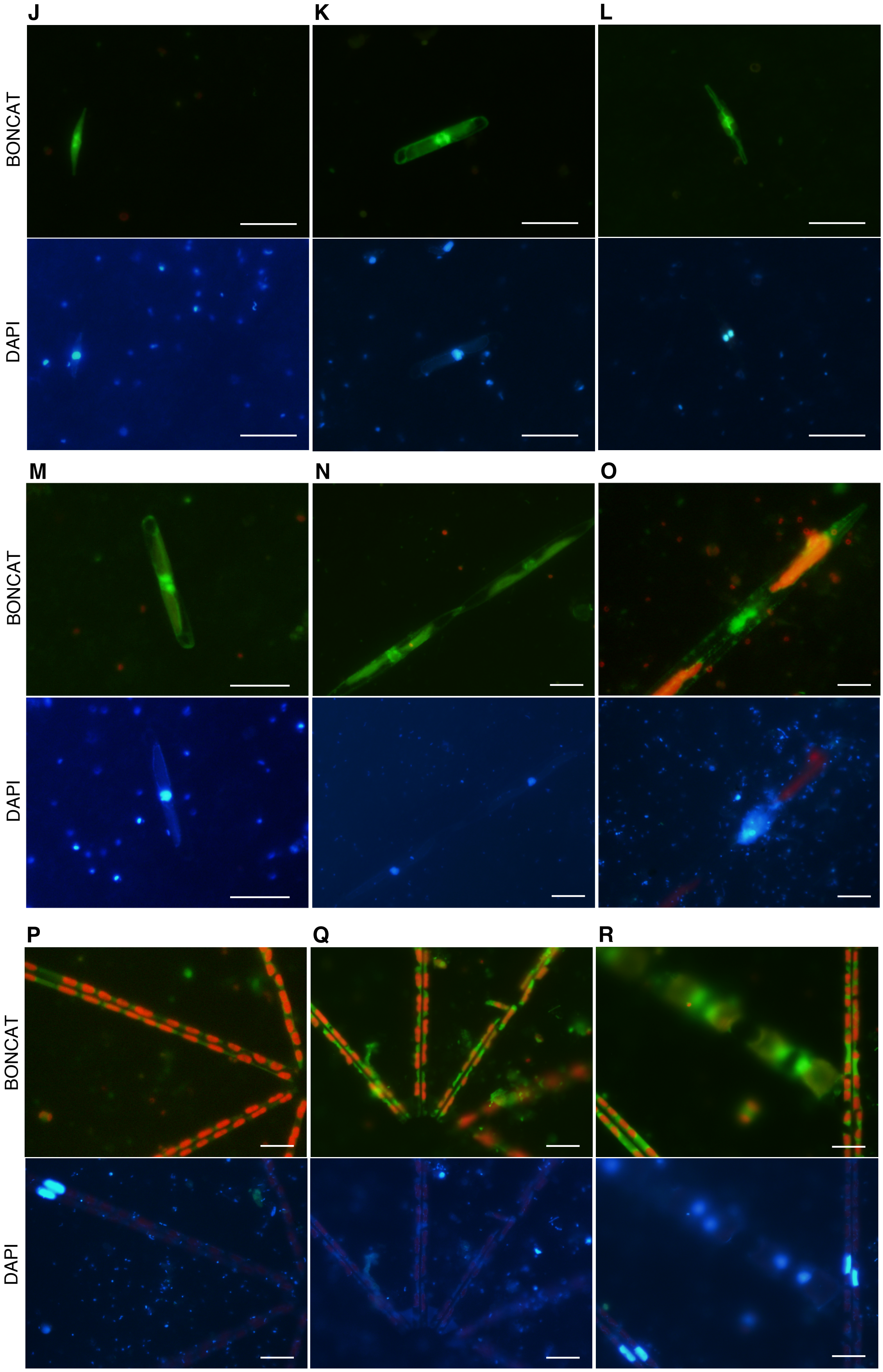

Centric diatoms

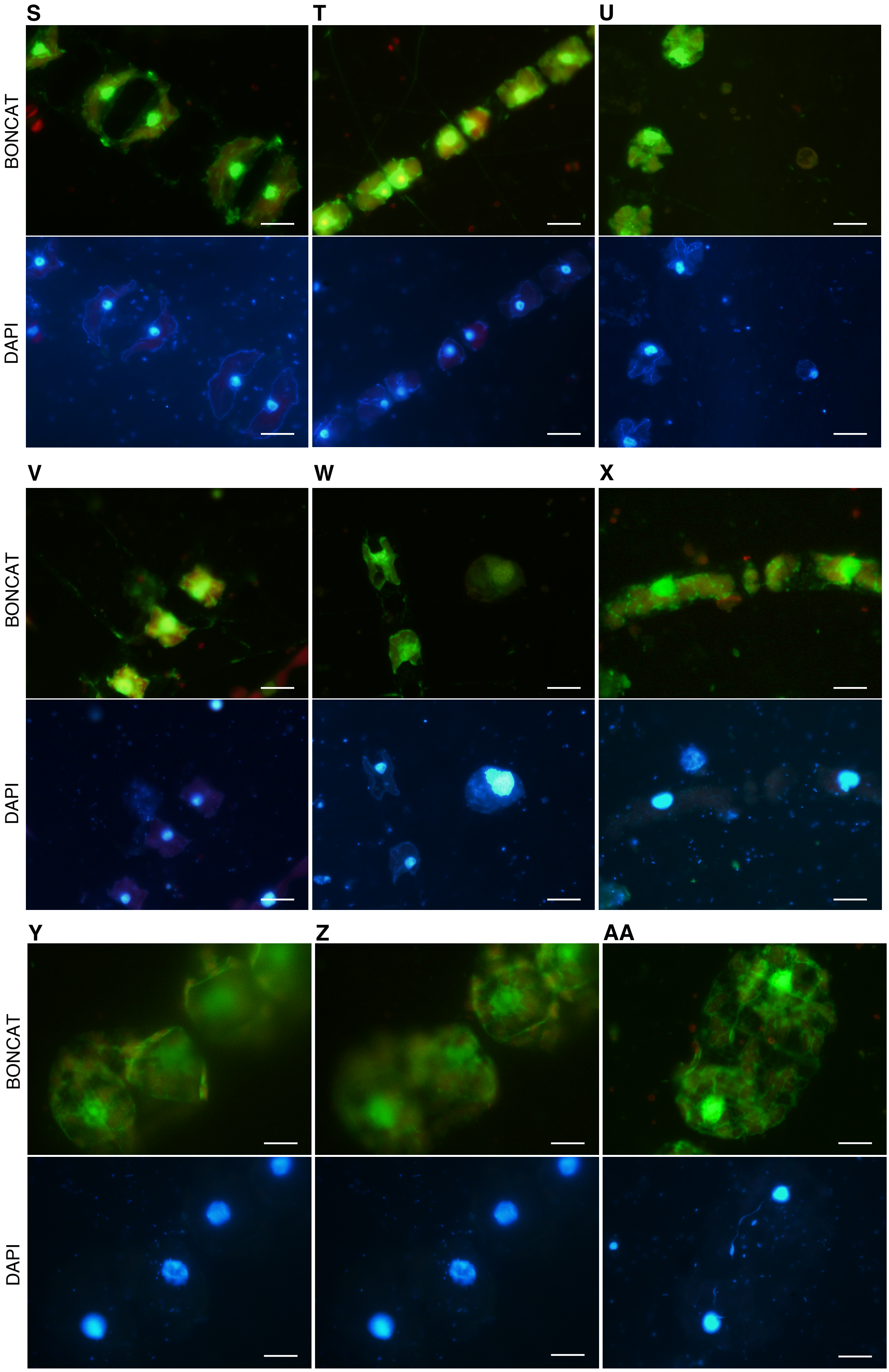

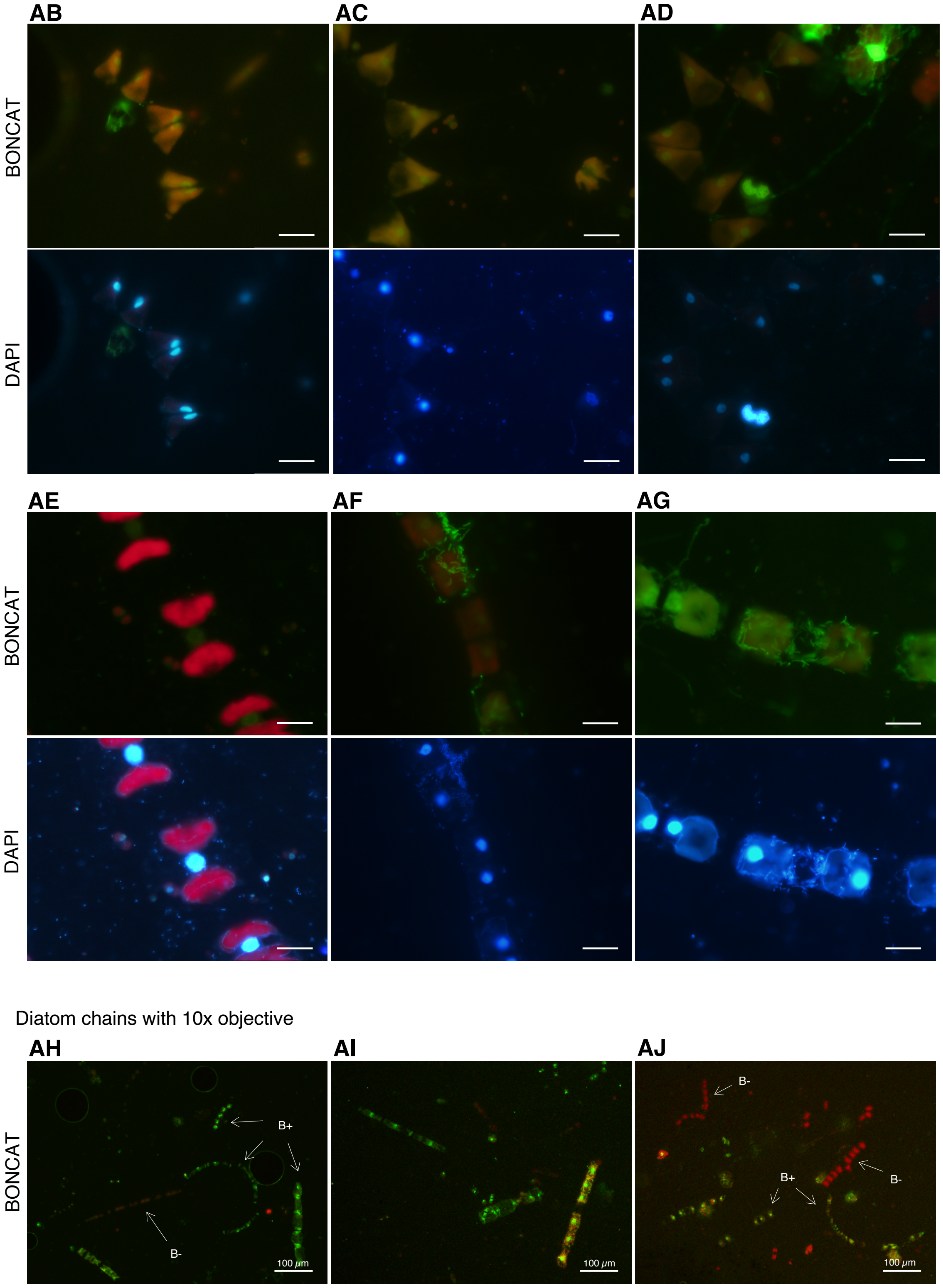

Figure S1. Microscopy images of BONCAT-positive cells of the different eukaryotic groups (see also Figure 1). Blue light (BONCAT) and UV light (DAPI) micrographs of eukaryotic cells. BONCAT fluorescence in bright green and chlorophyll fluorescence in red. Taxonomic identification to the genus level is indicated when possible, although we cannot confirm phylogenetic identity accurately. (**A-C**) Small eukaryotes (≤5 *µ*m). (**D-F**) Pigmented dinoflagellates, E and F images are the same cell with different focus. (**G-I**) Heterotrophic dinoflagellates (non-pigmented). BONCAT-positive structures are observed within dinoflagellate cells. (**J-R**) Pennate diatoms. (N) *Pseudo-nitzschia sp.* (P-R) Presumably *Thalassiotrix sp.*, found

always BONCAT-positive. (**S-AG**) Centric diatoms. (T and V) *Chaetoceros sp*. (Y-AA) Cells (unidentified genera) found always BONCAT-positive. (AB-AD) *Asterionellopsis sp.* cells, found always BONCAT-negative. (AE) Chain of BONCAT-negative diatom cells. (AF) Chain of BONCAT-negative diatom cells with associated BONCAT-positive bacteria. (AG) Chain of BONCAT-positive diatom cells with associated BONCAT-positive bacteria. (**AH-AJ**) Images taken with the 10x objective where large BONCAT-positive (B+) and negative (B-) diatom chains can be observed. Scale bar indicates 10 μm for all images except AH-AJ, where scale bar indicates 100 μm. All images were taken using an Olympus BX61 epifluorescence microscope.

FIGURE S2

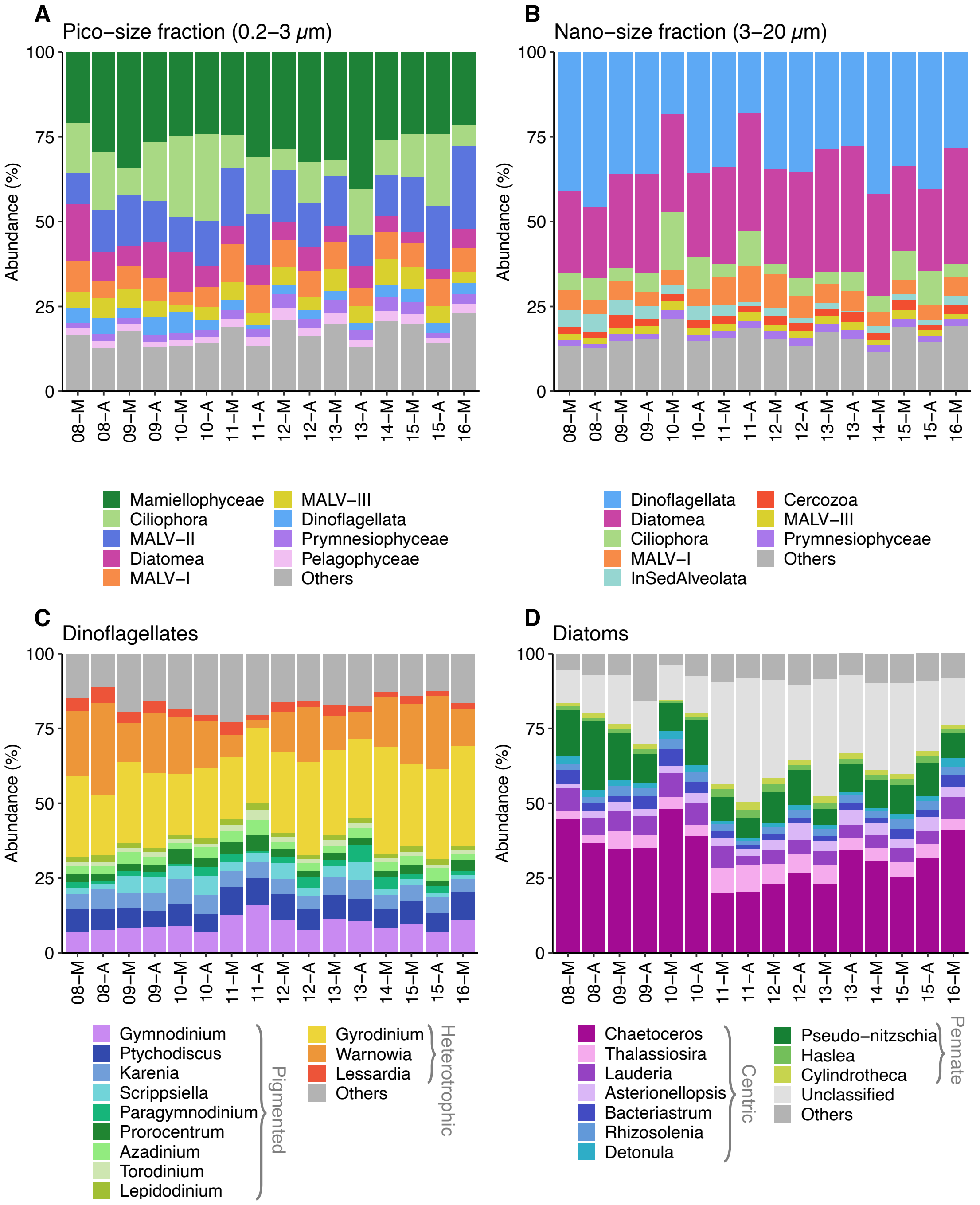

Figure S2. Eukaryotic community composition. Taxonomic composition, based on 18S rRNA gene sequencing, of the **(A)** pico- (0.2-3 *μ*m) and (**B**) nano-sized (3-20 *μ*m) fractions. Groups contributing ≤2% to total abundance are combined as 'Others'. Taxonomic composition of (**C**) dinoflagellates and (**D**) diatoms at the genus level. Groups contributing ≤1.5% to total abundance are combined as 'Others'. Dinoflagellate genera including species with chloroplasts are classified as pigmented in the legend. The 'Unclassified' group in (D) includes diatoms unclassified at the genus level. The x-axis indicates day-moment of the day (M: morning; A: afternoon).

FIGURE S3

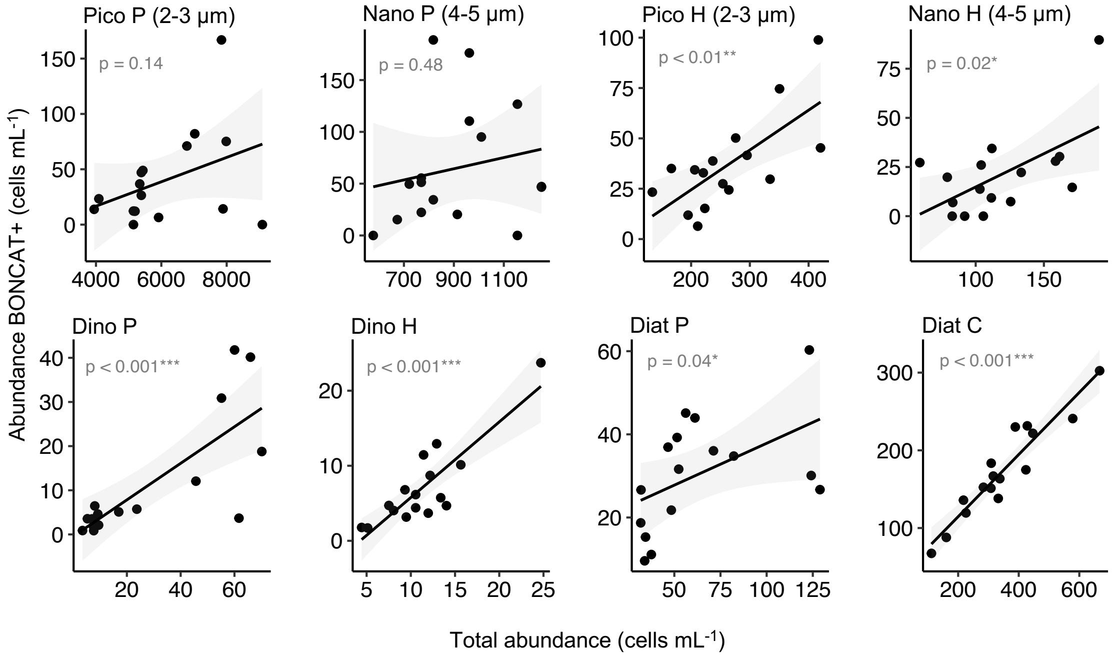

Figure S3. Correlation of BONCAT-positive and total cell abundance for the different eukaryotic groups. Black lines are linear regressions and shaded areas indicate the 95% confidence interval of the regression slope. The significance of the linear relationship (p value) is indicated for each correlation.

Figure S4

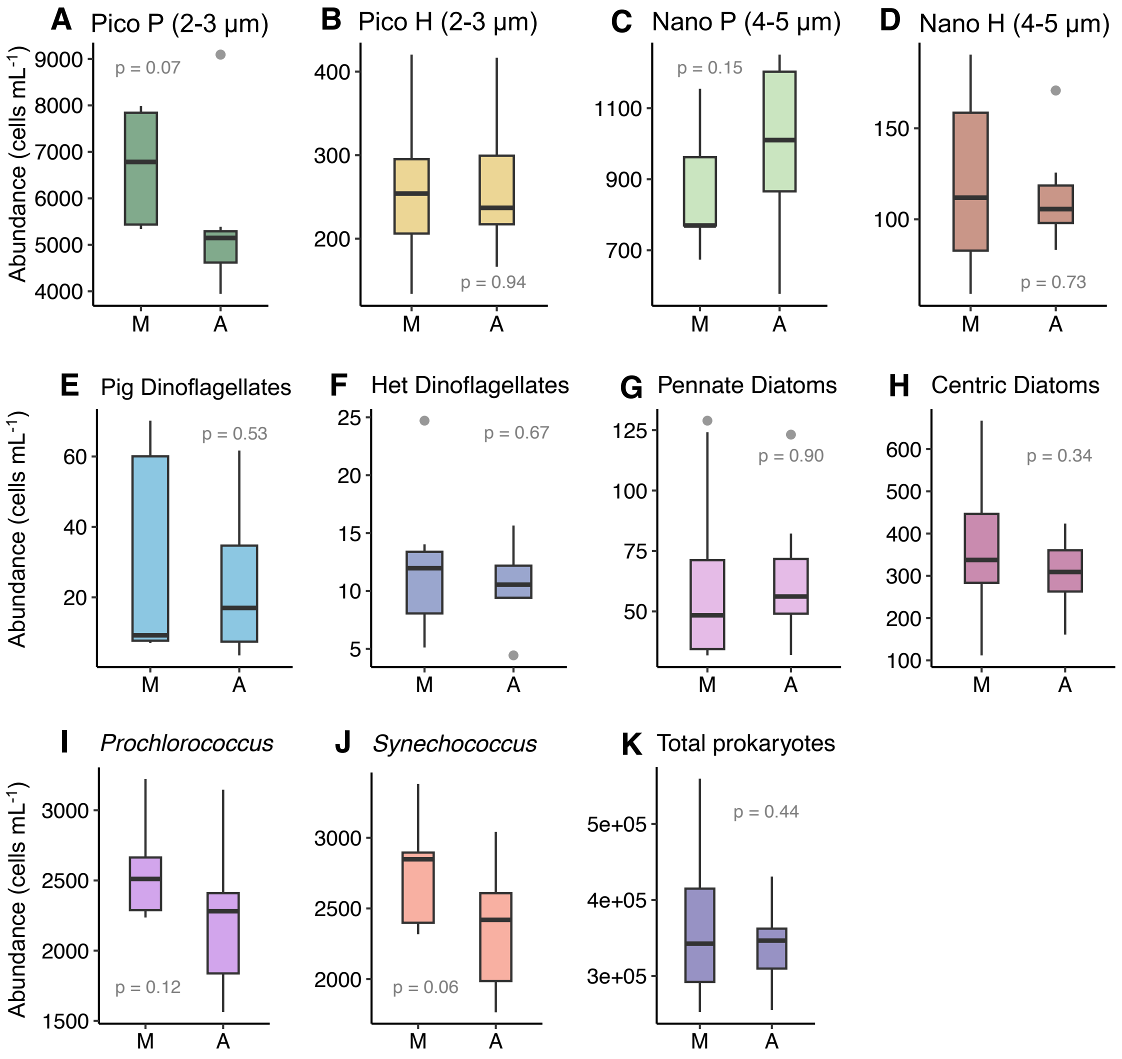

Figure S4. Morning *vs*. afternoon abundance variation for the different prokaryotic and eukaryotic groups. (A) Pigmented picoeukaryotes (Pico P, 2-3 *μ*m), (**B**) heterotrophic picoeukaryotes (Pico H, 2-3 *μ*m), (**C**) pigmented nanoeukaryotes (Nano P, 4-5 *μ*m), (**D**) heterotrophic nanoeukaryotes (Nano H, 4-5 *μ*m), (**E**) pigmented dinoflagellates, (**F**) heterotrophic dinoflagellates, (**G**) pennate diatoms, (**H**) centric diatoms, (**I**) *Prochlorococcus*, (**J**) *Synechococcus* and (**K**) total prokaryotes. Data beyond the end of the whiskers (outliers) are represented as grey dots in the boxplots. The x-axis indicates morning (M) and afternoon (A) samples. Significance (p value, Student's t-Test) of morning *vs*. afternoon variation is indicated for each boxplot.

FIGURE S5

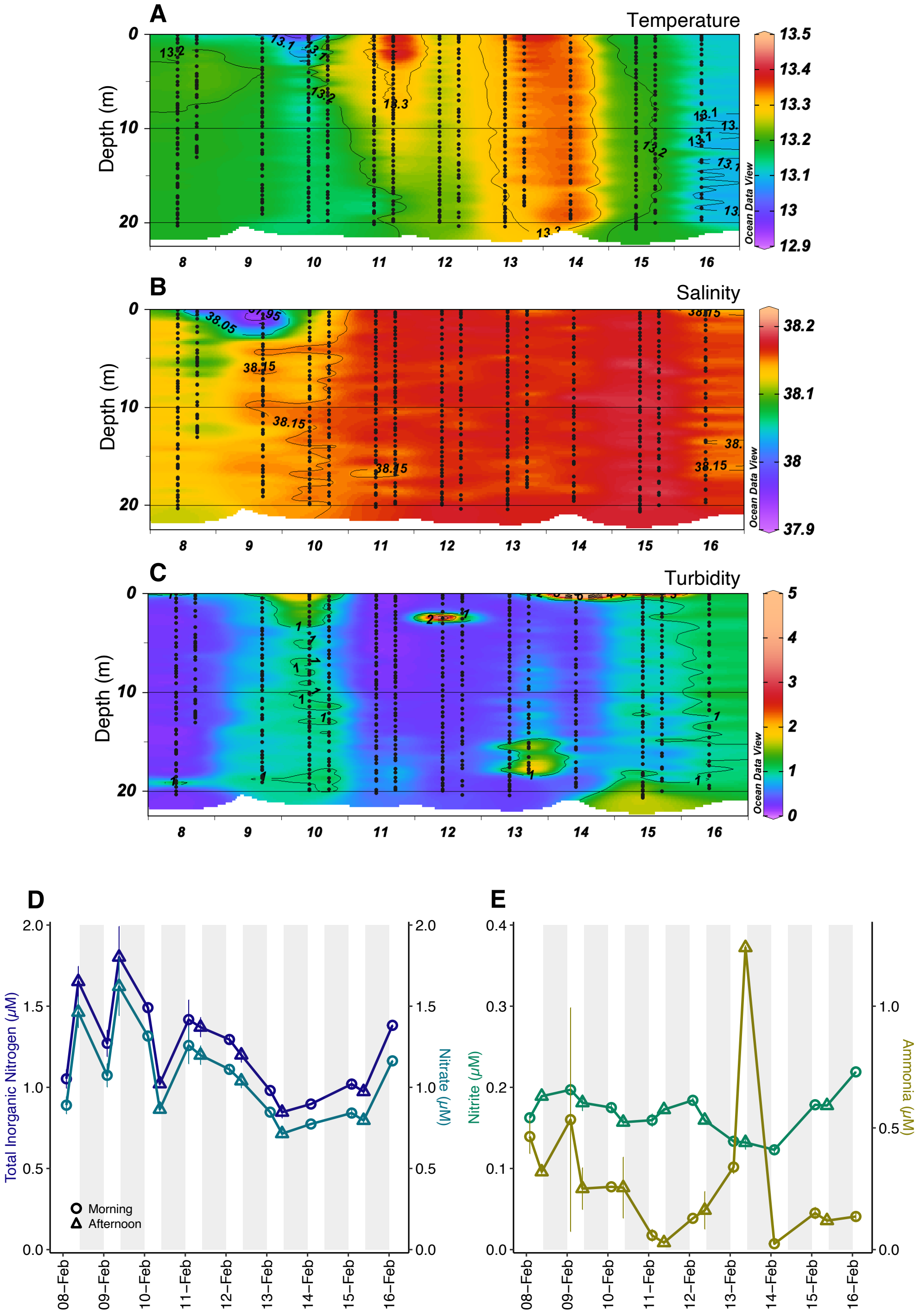
A
Temperature
Depth (m)
Ocean Data View
B
Salinity
C
Turbidity
D
Total Inorganic Nitrogen (μM)
Nitrate (μM)
Morning
Afternoon
E
Nitrite (μM)
Ammonia (μM)
08–Feb
09–Feb
10–Feb
11–Feb
12–Feb
13–Feb
14–Feb
15–Feb
16–Feb

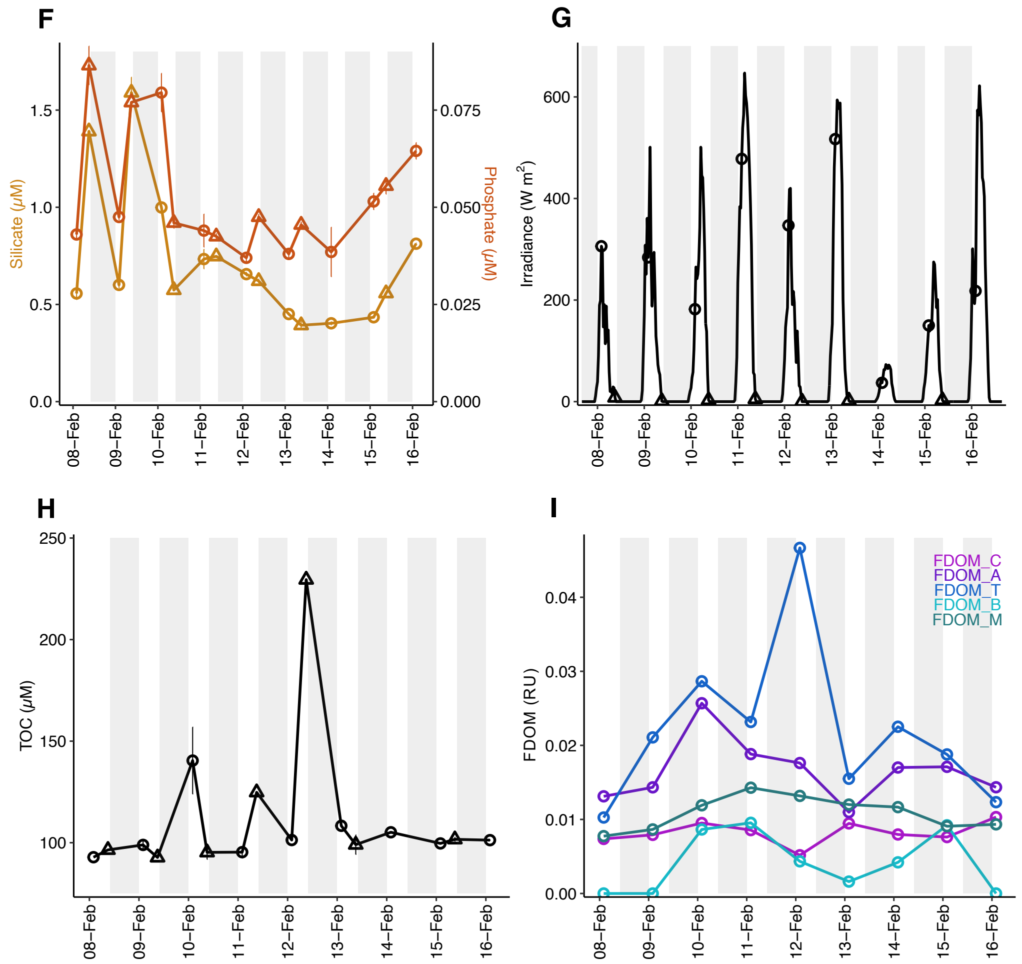

Figure S5. Environmental data over the sampling period. (**A**) Temperature (ºC), (**B**) salinity (g L^{-1}) and (**C**) turbidity (FTU) depth profiles. CTD downcast data are indicated by black dots. X-axis indicates the day of sampling (from 8th to 16th February). Values are interpolated using the weighted-average gridding from Ocean Data View 5.2.0 software. (**D-F**) Concentration of inorganic nutrients (μM): Total inorganic nitrogen, nitrate, nitrite, ammonia, silicate and phosphate. (**G**) Global solar irradiance (W m^2). (**H**) Concentration of total organic carbon (TOC, μM). (**I**) Fluorescent dissolved organic matter (FDOM) in Raman Units (RU), note that measurements are only available for morning samplings. In D-I, white-grey areas indicate day-night periods, x-axis indicate sampling day (day-month), and circles and triangles indicate morning and afternoon samplings, respectively. Vertical error bars in D-F and H show standard deviation of two replicates.

FIGURE S6

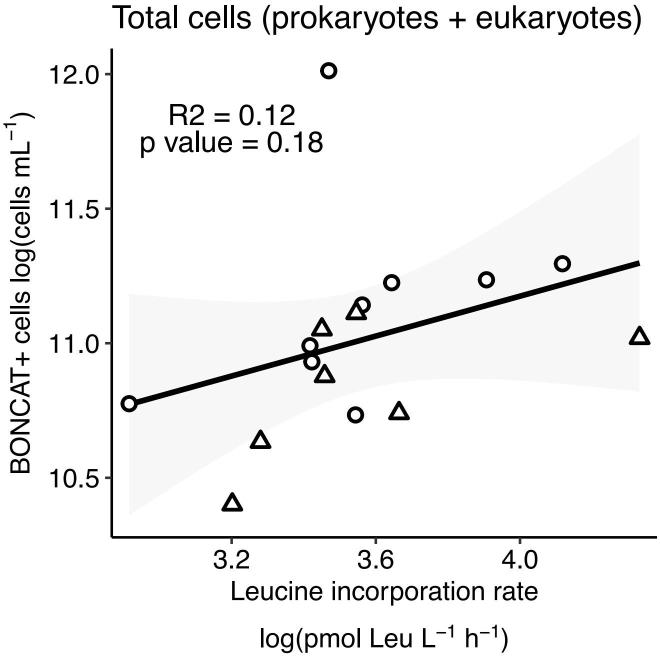

Figure S6. Relationship between total BONCAT-positive cell abundances (prokaryotes and eukaryotes) and bulk ^{3}H-leucine incorporation rates. The R^2 and p value is indicated for the linear model. Shaded grey area indicates the 95% confidence interval of the regression slope. Circles and triangles indicate morning and afternoon samplings, respectively.

FIGURE S7

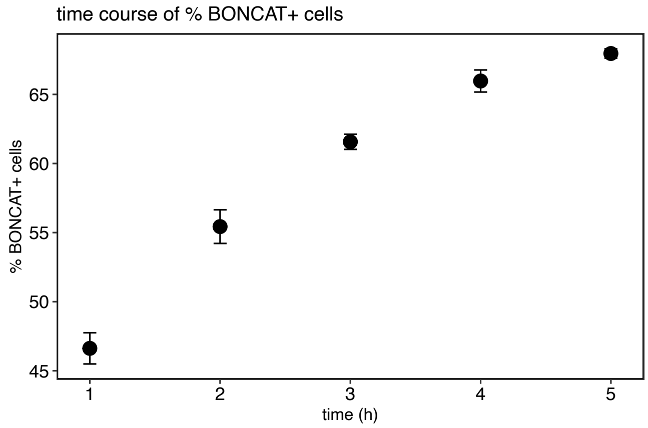

Figure S7. Time course experiment with BONCAT-positive bacteria. Percentage of BONCAT-positive bacteria with time during 5 h of incubation. This experiment was performed with bacterial communities from the Blanes Bay and is part of the study published by Leizeaga et al. (2017) in Frontiers in Microbiology.

FIGURE S8

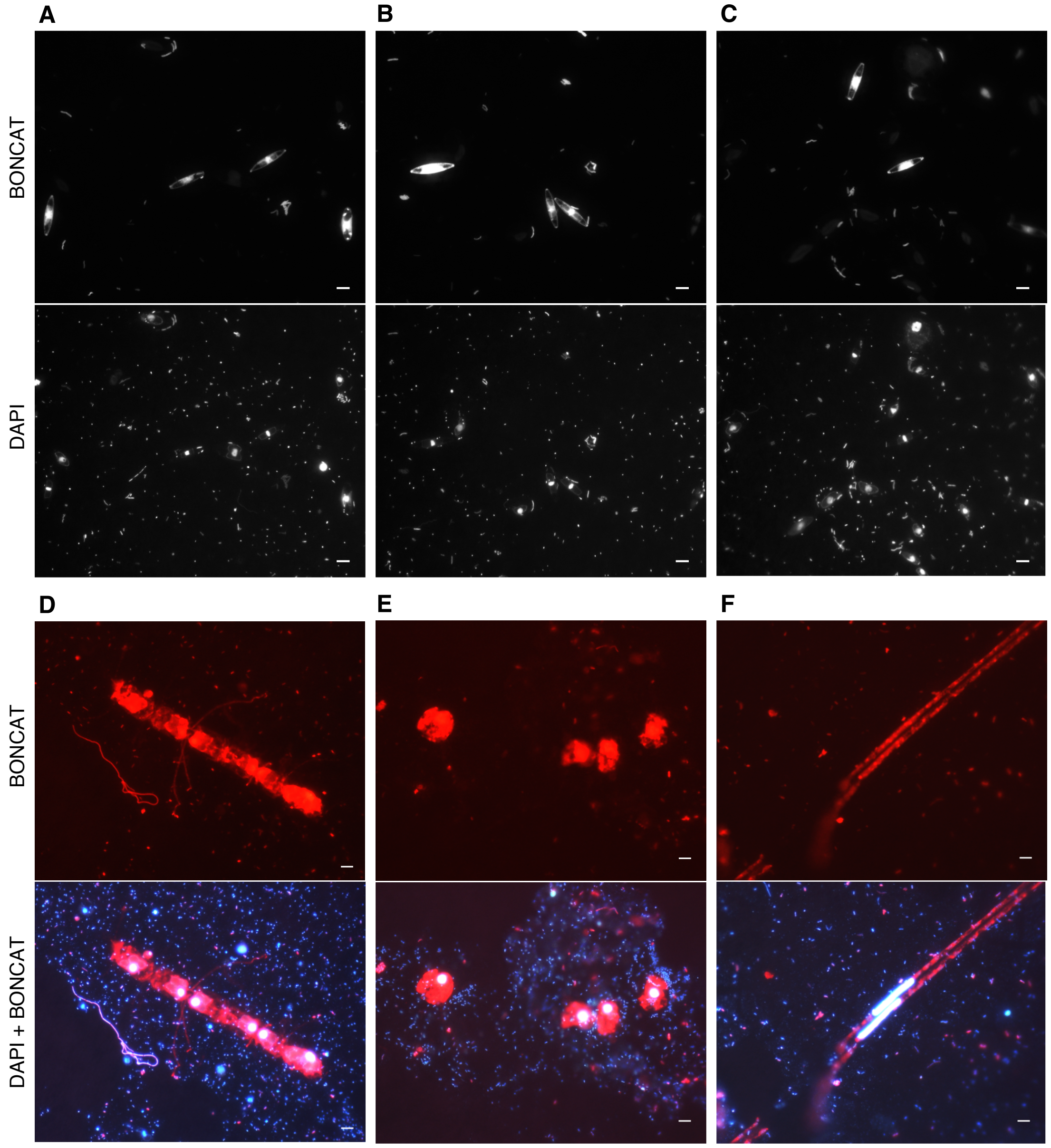

Figure S8. Microscopy images of BONCAT-positive eukaryotic cells from other marine sites (unpublished data). (**A-C**) Samples from the Mar Menor (Murcia, Spain), a eutrophic coastal saline lagoon of the Mediterranean Spanish coast, showing strongly labelled pennate diatoms. Samples were taken in April 2023 between 4 to 18 m distance to the shore, in a site highly influenced by mining and agricultural anthropogenic pressures (Mena et al., unpublished data). For the BONCAT click reaction, the CR110 azide dye was used (blue excitation, green emission). Black and white micrographs show cells fluorescence under blue light (BONCAT) and UV light (DAPI). (**D-F**) Samples from a mesocosm experiment conducted in the Eastern North Atlantic oligotrophic waters in November 2018 (Gómez-Letona et al., unpublished data), showing strongly labelled centric and pennate diatoms. For the BONCAT click reaction, the Alexa594 azide dye was used (orange excitation, red emission). Colored micrographs show cells fluorescence under red light (BONCAT) and under both UV and red light (DAPI + BONCAT). Images were acquired using a Zeiss Axio Imager epifluorescence microscope connected to a black and white (in case of A-C images) or a color (in case of D-F images) Zeiss camera (AxioCam MRm) at 630x magnification. Scale bar indicates 5 μm.

SUPPLEMENTARY TABLES

Table S1. Redundancy analysis (RDA) results of the BONCAT-positive eukaryotic community. The function 'rda' from the R package was used for the calculations. For the model, 999 permutations were used, and only non-collinear variables were included. The estimated degrees of freedom (Df), variance (Var), F statistic and probability (p value) are indicated. The asterisk indicates significance level $p < 0.05$ and no asterisk $p > 0.05$. The variable 'Time' indicates morning *vs*. afternoon, 'PROK' indicates prokaryotic abundance and 'TOC' indicates total organic carbon. Results depicted in Fig. 5A in the manuscript.

	Df	Var	F	p value
**Axes**				
RDA1	1	599.20	4.8501	0.542
RDA2	1	303.11	2.4534	0.951
**Constrained variables**				
Time	1	431.48	2.9105	0.034 *
Irradiance (IR)	1	162.11	1.0935	0.369
Prok abundance (PROK)	1	124.74	0.8414	0.519
NH4	1	109.80	0.7406	0.602
Day	1	96.88	0.6535	0.645
NO3	1	79.61	0.5370	0.742
Turbidity (Tur)	1	72.17	0.4868	0.786
NO2	1	37.33	0.2518	0.932
TOC	1	13.57	0.0916	0.992
Residual	*5*	*741.26*		

Table S2. Multiple regression model results between leucine incorporation rates and BONCAT-positive eukaryotic cell abundances. ANOVA table of the regression model computed using the functions 'lm', 'summary.lm' and 'anova' from the R package. The regression model includes leucine incorporation rates as the continuous dependent variable and all the eukaryotic groups and prokaryotes in terms of B+ cell abundances as predictor variables. The estimated degrees of freedom (Df), sum of squares (Sum Sq), F value and significance (p value) are indicated. The proportion of variance in leucine incorporation rates explained by each group (Var Exp %) is calculated by dividing the Sum Sq value of each group by the total sum of squares. The asterisks indicate significance level $p < 0.01$ and no asterisk $p > 0.05$. The multiple R^2 and adjusted R^2 of the regression model is 0.83 and 0.57, respectively.

	Df	Sum Sq	F value	p value	Var Exp (%)
Centric diatoms	1	1.097	22.341	0.003 **	63.14
Heterotrophic dinoflagellates	1	0.1288	2.625	0.156	7.42
Pigmented dinoflagellates	1	0.0093	0.189	0.678	0.53
Pigmented nanoeukaryotes	1	0.1142	2.325	0.178	6.57
Prokaryotes	1	0.0096	0.197	0.672	0.55
Pennate diatoms	1	0.0213	0.435	0.534	1.23
Heterotrophic picoeukaryotes	1	0.0261	0.533	0.492	1.51
Pigmented picoeukaryotes	1	0.0359	0.732	0.425	2.07
Heterotrophic nanoeukaryotes	1	0.0001	0.003	0.952	0.01
Residual	*6*	*0.2946*			*16.95*
